# Effective Elasticity of Anatase-Derived TiO_2_ Nanoclusters: Volume Normalization, Directional Response, and Structure–Property Correlations

**DOI:** 10.3390/nano16211382

**Published:** 2026-10-28

**Authors:** Robert M. Solomon, Corneliu I. Oprea, Mihai A. Gîrțu

**Affiliations:** 1 Doctoral School of Physics, Faculty of Physics, University of Bucharest, 077125 Bucharest, Romania; robert-mihai.solomon@s.unibuc.ro; 2 Faculty of Applied Sciences and Engineering, Ovidius University of Constanța, 900527 Constanța, Romania; cornel.oprea@univ-ovidius.ro

**Keywords:** anatase TiO_2_, nanoclusters, effective elastic coefficients, reference volume, elastic anisotropy, morphology-dependent elasticity, surface coordination, energy–strain method, density functional theory

## Abstract

Elastic response affects TiO_2_ nanostructure durability and performance and depends on particle size and morphology. We determine effective elastic coefficients, moduli, and anisotropy indices from clamped-ion density functional theory energy–strain calculations for nine anatase-derived clusters containing 10–165 Ti atoms, using a tetragonal-form model. Comparing framework convex-hull, hydroxyl-inclusive convex-hull, and bulk-equivalent volumes shows that normalization can create or reverse apparent size trends. Across all five matched parent–cut pairs, cutting and passivating the apices reduces axial stiffness relative to in-plane stiffness and increases relative in-plane coupling and shear. These directional changes are independent of the volume convention. Cutting removes asymmetric axial Ti environments, while hydroxylation satisfies local coordination without restoring the removed framework connectivity. After cutting, mean Ti–O distances shorten, their dispersion narrows, and the mean bond order around 4-coordinate Ti increases, whereas axial stiffness decreases relative to in-plane stiffness. Thus, local bond strengthening coexists with reduced relative axial stiffness. The frontier-orbital gap widens with increasing 4-coordinate Ti fraction and shorter mean Ti–O bonds, while coordination-resolved electronic descriptors also track effective stiffness and anisotropy. These descriptor associations remain exploratory because size, morphology, coordination, and passivation are coupled. Meaningful comparisons of nanocluster elasticity must therefore specify the volume convention and distinguish local bond strengthening from changes in directional stiffness.

## 1. Introduction

Titanium dioxide is one of the most extensively studied metal oxides [1], owing to its widespread use in paints [2] and coatings [3] with antibacterial, anticorrosion, anti-fogging, or self-cleaning properties [4], polymers [5] and rubber composites [6], in sensor electronics for detection of gases, chemicals [7], or biological materials [8] and micro- and nano-electromechanical devices [9], in photocatalytic degradation of pollutants [10,11,12] as well as in dye-sensitized solar cells, for energy conversion [13,14,15]. Mechanical properties such as stiffness, elastic stability, and resistance to deformation are relevant to the structural integrity and durability of TiO_2_-based materials [16]. Experimental measurements of shape-dependent compressibility in anatase nanoparticles demonstrate that their mechanical response depends on particle geometry [17].

Titanium dioxide has four polymorphs in nature: tetragonal (anatase), tetragonal (rutile), orthorhombic (brookite), and monoclinic (TiO_2_-B) [18,19], as well as two high-pressure forms synthesized from the rutile phase. Rutile is the most thermodynamically stable polymorph of TiO_2_ in the bulk, whereas anatase is very common and stable in nanomaterials [20,21], displaying the highest photocatalytic activity [22]. As anatase is the most interesting phase of TiO_2_ for many applications, it will be the focus of our interest in the following.

In most practical applications, such as photocatalysis, photovoltaics, sensing, protective coatings, etc., titanium dioxide is not used in bulk, but as finite, surface-rich, faceted, and often truncated or reconstructed nanoparticles [23]. These are the preferred forms because the large surface-to-volume ratio amplifies reactivity and charge-transfer efficiency [22]. The nanoparticles’ properties are controlled not only by composition and size, but also by morphology and surface coordination [1,24].

Mechanical properties matter alongside thermal, optical and electronic response. Interfacial stresses at nanoparticle–matrix contacts determine load transfer and cohesion in composite films. Strain-coupling between mechanical deformation and electronic structure modifies photocatalytic and photoelectrochemical activity, and the stability of individual particles under compression sets practical limits on device durability [25]. Characterizing the elastic properties of TiO_2_ nanoparticles is therefore a prerequisite for rational materials design, yet this characterization remains incomplete [26].

The mechanical properties of anatase TiO_2_ have been studied for the bulk using ab initio calculations such as density functional theory (DFT) [27,28,29,30,31,32,33], density functional perturbation theory [34], and DFT augmented with an empirical dispersion term [35]. DFT strategies and methodological developments for doped TiO_2_ have also been reviewed [36]. However, despite the numerous computational studies [24], the elastic properties of nanostructured TiO_2_ have not been sufficiently explored. Molecular dynamics simulations have examined the elastic properties of TiO_2_ nanotubes and nanowires (i.e., the Young’s modulus, Poisson ratio, and shear modulus) [37] and size-dependent elastic modulus models based on surface-bond contraction have been applied to TiO_2_ nanocrystals [38], but these approaches do not provide the complete tetragonal tensor.

Although atomically precise clusters, such as (TiO_2_)*_n_* clusters with *n* = 16 to 68 [39], 14 to 54 [40], 32 to 122 [41], 33 to 132 [42], and 38 to 411 [43,44] have been widely used as model systems to understand electronic confinement and surface reactivity, mechanical properties and, in particular, how elastic stiffness evolves with cluster size, morphology, and surface termination is still largely unknown. Unlike periodic crystals, finite clusters do not admit a rigorous definition of elastic coefficients in the continuum sense, as stress and strain are not uniquely defined without translational symmetry [38,45,46]. Nevertheless, valuable insight into their mechanical response can be obtained from the curvature of the total energy with respect to controlled deformations [47,48]. When treated consistently, such energy–strain relationships can provide effective elastic coefficients that allow meaningful comparisons across sizes, morphologies, and surface chemistries.

Among the various TiO_2_ nanostructures, anatase bipyramidal [49] and octahedral [50] clusters have attracted particular attention as realistic models of nanocrystals exposing low-energy facets [24,51]. The thermodynamically preferred equilibrium morphology of anatase is a slightly truncated tetragonal bipyramid exposing {101} facets on the lateral faces and a small fraction of {001} facets at the truncated tips [51,52]. Hydrothermal and solvothermal synthesis protocols can tune the relative area of {001} facets from a few percent to the majority of the surface, producing nanocrystals that span the range from elongated bipyramids to flat platelets [22,53,54]. Since {101} and {001} surfaces differ in their atomic structure, coordination environment, and reactivity, the photocatalytic and photoelectrochemical performance of anatase nanoparticles depends strongly on particle shape as well as size [55,56].

Previous first-principles studies have shown that the apical regions of such particles host highly undercoordinated Ti sites that are associated with enhanced chemical reactivity [52,57]. Systematic truncation of these apical motifs therefore provides a controlled way to modify both the local coordination environment and the overall particle morphology, enabling the investigation of how specific structural features influence macroscopic-like properties such as elastic response [58,59]. A central challenge in modeling finite TiO_2_ clusters is the treatment of surface termination. Bare, bulk-truncated clusters often contain unrealistically undercoordinated atoms and can undergo significant reconstruction or charge localization [60]. To address this issue and ensure consistent chemical boundary conditions, surface passivation is commonly employed [52].

Here we use first-principles calculations to determine effective elastic coefficients for nine optimized anatase TiO_2_ nanoclusters containing *n* = 10–165 Ti atoms. We compare stoichiometric bipyramidal clusters, which reproduce the equilibrium {101}-faceted anatase habit, with hydroxyl-passivated structures obtained by removing two or four atomic layers from the apical regions of the bipyramidal parents. This construction allows size trends to be followed within the three morphology series and enables matched parent–cut comparisons of apical-layer removal. All clusters were fully optimized with density functional theory, and the six independent effective coefficients were extracted from total-energy calculations under small, controlled deformations by fitting the energy–strain curves to quadratic forms. Converting those curvatures into stiffness units, however, requires a reference volume, and a finite cluster has no unique one. We therefore report the coefficients under three conventions: a composition-based bulk-equivalent volume, the convex hull of the TiO_2_ framework, and the convex hull of the whole hydroxylated structure. We treat the bulk-equivalent volume as primary, since it assigns the same anatase volume to every TiO_2_ unit and so introduces no geometric factor that itself varies with size, shape or passivation.

Against this background, the present study makes two related contributions: an analysis of directional effective elasticity in the specified anatase-derived cluster series, and a comparison of reference-volume conventions applied to the same energy–strain data. Three linked questions are addressed. First, which apparent size trends arise from the energy–strain response itself, and which are introduced by the reference volume used to express that response in pressure units? Second, how does the combined cutting-and-passivation intervention change the directional response? Third, do the accompanying changes in coordination, bonding, and electronic structure translate into uniform stiffening, or can stronger local bonds coexist with reduced relative axial stiffness?

The matched parent–cut construction allows the accompanying structural, electronic, and elastic changes to be examined within each parent family. We show that the volume convention can reverse apparent scalar stiffness trends, whereas apical cutting consistently shifts stiffness from the axial direction toward in-plane coupling and shear. At the same time, cutting produces shorter, more regular, and electronically stronger local Ti–O bonds while reducing axial stiffness relative to in-plane stiffness. This leads to the central physical proposition examined here: local coordination and bond strength describe the chemistry of individual sites, whereas the position, orientation, and connectivity of those bonds determine the directional elasticity of the whole nanocluster.

## 2. Methods

This section describes the construction of the anatase TiO_2_ clusters, the deformation-based extraction of effective elastic coefficients, the reference-volume conventions and comparative analyses, and the computational details.

### 2.1. Construction of Anatase TiO_2_ Clusters

Finite anatase TiO_2_ nanoclusters were constructed starting from the bulk anatase crystal structure as bipyramidal fragments exposing low-energy facets. The parent clusters adopt a morphology consistent with Wulff constructions of anatase nanocrystals, dominated by {101} facets and terminated by apical regions along the crystallographic [001] direction.

**Figure 1:**
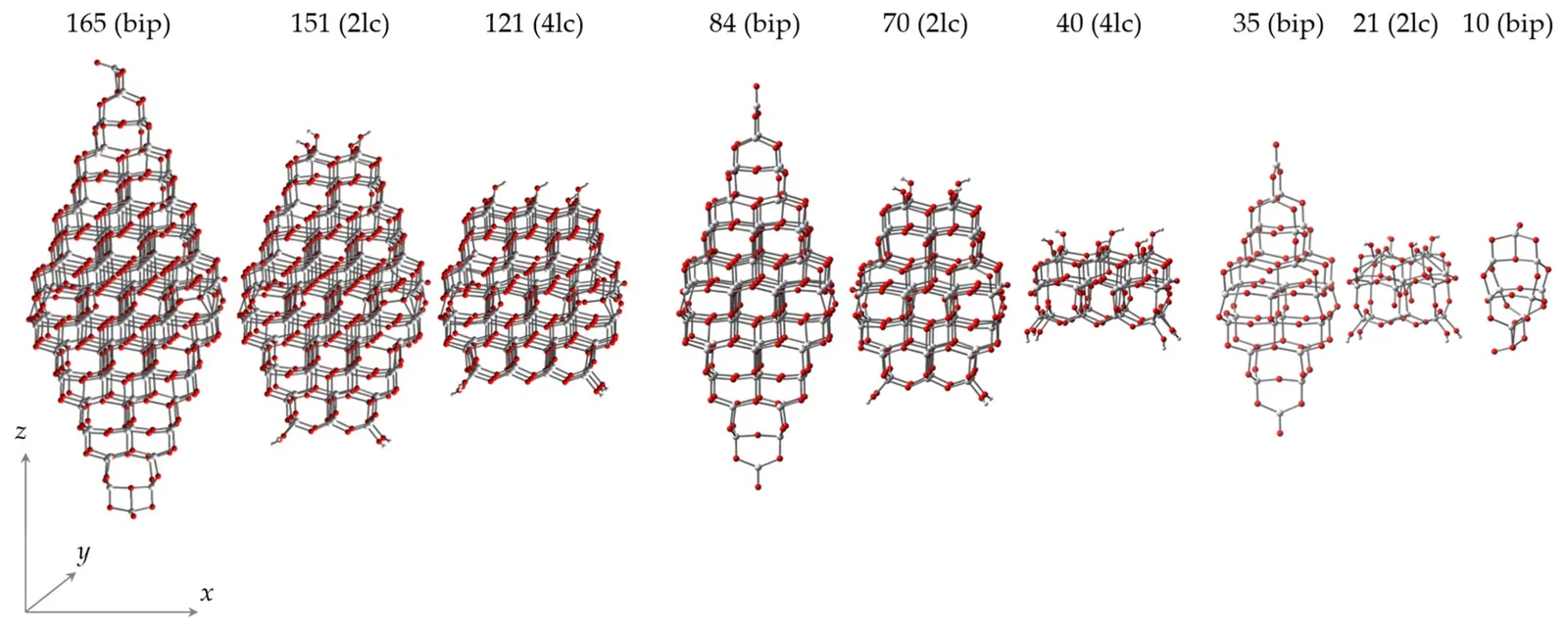
Equilibrium structures of the anatase-derived TiO_2_ clusters used as the unstrained reference configurations. Structures are grouped by families, according to their bipyramidal parent, to show the effect of layer removal, and rescaled for visibility. The labels give the number of Ti atoms and the cluster type: bipyramidal (bip), 2-layer cut (2lc), or 4-layer cut (4lc). The cut structures were hydroxylated after removal of the apical layers. The *z* axis is aligned with the long axis of each bipyramidal parent, and the *x* and *y* axes define the transverse plane. Ti, O, and H atoms are shown in dark gray, red, and light gray, respectively.

Cluster size is defined by the number of Ti atoms *n*. The four bipyramidal clusters (*n* = 10, 35, 84, and 165 Ti atoms) serve as the primary size series. They represent small, intermediate, and relatively large nanocrystals within the computationally accessible regime. Their coordinate-based dimensions, ranging from 0.48 to 3.93 nm across the three Cartesian directions, are reported in Table S2. The long axis of each bipyramidal cluster coincides approximately with the anatase [001] direction, while the transverse directions correspond to the [100] and [010] crystallographic axes. Their elongation along the crystallographic z axis reflects the large c/a ratio of anatase and is clearly visible in Figure 1 across all four sizes.

The five cut clusters were obtained by removing two or four consecutive atomic layers perpendicular to *z* from the three larger bipyramidal parents, from both apical regions along the long axis. The apical Ti layers were identified as the outermost Ti atoms along the [001] direction and removed symmetrically from the top and bottom of the cluster (preserving the mirror symmetry about the transverse plane that the shear-deformation analysis relies on) together with oxygen atoms belonging to the same apical structural motif when required to avoid chemically detached species. This truncation procedure resulted in the 2-layer cut series (*n* = 151, 70, 21) and the 4-layer cut series (*n* = 121, 40). Cutting reduces the *z*-axis extent while leaving the in-plane cross-section largely unchanged, making bipyramidal and cut clusters of similar Ti count geometrically distinct rather than simply different in size.

The composition of the bipyramidal nanoclusters is stoichiometric, (TiO_2_)*_n_*. The 2-layer cut clusters were hydroxylated (to ensure chemically realistic surface termination) with 8 –OH groups, giving the formula Ti*_n_*O_2*n*_(H_2_O)_4_. The 4-layer cut clusters were hydroxylated with 12 –OH groups, resulting in Ti*_n_*O_2*n*_(H_2_O)_6_. Hydroxylation was performed by adding surface oxygen and hydrogen atoms to undercoordinated surface sites following a consistent coordination-based scheme, restoring near-bulk Ti–O coordination while preserving the underlying anatase structure. For each cluster, several initial hydroxyl orientations were tested, and the lowest-energy configuration was retained for subsequent calculations.

The equilibrium cluster geometries used as the unstrained reference structures are shown in Figure 1. The systems are arranged according to their parent–cut relationships. The *n* = 165 parent gives the *n* = 151 and *n* = 121 structures after removal of two and four atomic layers, respectively. The *n* = 84 parent gives the *n* = 70 and *n* = 40 structures, whereas the *n* = 35 parent gives the *n* = 21 structure after removal of two layers. The small *n* = 10 bipyramidal cluster has no corresponding cut structure. This grouping defines the paired comparisons used in the subsequent analysis.

The *n* = 10, 35, 84, and 165 bipyramidal parent sizes match the structures and sizes of the {101}-faceted, octahedral-symmetry (O_h_) TiO_2_ nanoclusters studied earlier by another group [59]. They used the same Wulff-construction principle to build a series extending up to *n* = 455 and examined how structural parameters (Ti–O bond length, Ti and O coordination number) and electronic-structure properties converge toward the bulk anatase limit with increasing size. Using an independent, all-electron relativistic DFT approach, they found the average Ti–O distance and coordination number to approach the bulk values only gradually over this size range, still short of full convergence at *n* = 165, while genuinely bulklike electronic behavior was not reached until *n* larger than 15,000, when the same B3LYP functional was used [59], roughly two orders of magnitude greater than the largest cluster considered here. The present bipyramidal series therefore samples structures from the same well-characterized part of the size-convergence curve, still well short of bulk in both structure and electronic character, consistent with the departures from bulk elastic behavior reported in Section 3.3 and with the coordination-dependent electronic descriptors examined in Section 3.7.

### 2.2. Determination of Elastic Coefficients from Finite Deformations

The elastic response of the clusters was evaluated by computing the curvature of the total energy with respect to a set of small, controlled deformations. In the linear elastic regime, stress and strain are related by Hooke’s law, Equation (1), with the elastic stiffness tensor *C_ij_*. The elastic coefficients are equivalently defined as the second derivatives of the total energy with respect to strain, Equations (2) and (3), which is the form used in the present energy–strain approach.

(1) $$\sigma_{i}=\sum_{j}C_{\mathrm{ij}}\varepsilon_{j}$$

The strain can be expressed using the Voigt notation [61] ***ε*** = (*ε*_1_,*ε*_2_,*ε*_3_,*ε*_4_,*ε*_5_,*ε*_6_), where ε_1_ = ε_xx_, ε_2_ = ε_yy_, ε_3_ = ε_zz_ are normal strains and ε_4_ = 2ε_yz_, ε_5_ = 2ε_xz_, ε_6_ = 2ε_xy_ are shear strains. The elastic coefficients *C_ij_* are second order elastic coefficients, obtained from the Taylor expansion of the total energy of the solid in terms of strain components [47,48]:

(2) $$E\left(V,\left\{\varepsilon_{i}\right\}\right)=E\left(V_{0},0\right)+V_{0}\sum_{i}\sigma_{i}\varepsilon_{i}+\frac{1}{2}V_{0}\sum_{i,j}C_{\mathrm{ij}}\varepsilon_{i}\varepsilon_{j}$$

where *V*_0_ and *V* are the volume of the solid at equilibrium and under deformation, respectively, such that:

(3) $$C_{\mathrm{ij}}=\frac{1}{V_{0}}{\left.\frac{\partial^{2}E}{\partial \varepsilon_{i}\partial \varepsilon_{j}}\right|}_{0}$$

Although the elastic coefficients are rigorously defined only for periodic solids, applying bulk-inspired deformation patterns to finite clusters allows the extraction of *effective* elastic coefficients from the curvature of the total energy with respect to strain.

The elastic response of a material of arbitrary symmetry is represented by a symmetric 6 × 6 stiffness matrix containing up to 21 independent coefficients. Bulk anatase TiO_2_ belongs to the tetragonal point group D_4_h (4/mmm), for which the stiffness tensor has six independent coefficients: *C*_11_, *C*_12_, *C*_13_, *C*_33_, *C*_44_, and *C*_66_ [62]. For the finite clusters, exact tetragonal symmetry is not assumed to be rigorously preserved. We nevertheless use the corresponding six-coefficient matrix as an effective model in the cluster-fixed reference frame:

(4) $$C=\left(\begin{array}{ccc} \begin{array}{ccc} C_{11} & C_{12} & C_{13}\\ C_{12} & C_{11} & C_{13}\\ C_{13} & C_{13} & C_{33} \end{array} & \begin{array}{c} 0\\ 0\\ 0 \end{array} & \begin{array}{c} \begin{array}{cc} 0 & 0 \end{array}\\ \begin{array}{cc} 0 & 0 \end{array}\\ \begin{array}{cc} 0 & 0 \end{array} \end{array}\\ \begin{array}{ccc} 0 & 0 & 0 \end{array} & C_{44} & \begin{array}{cc} 0 & 0 \end{array}\\ \begin{array}{ccc} \begin{array}{c} 0\\ 0 \end{array} & \begin{array}{c} 0\\ 0 \end{array} & \begin{array}{c} 0\\ 0 \end{array} \end{array} & \begin{array}{c} 0\\ 0 \end{array} & \begin{array}{cc} \begin{array}{c} C_{44}\\ 0 \end{array} & \begin{array}{c} 0\\ C_{66} \end{array} \end{array} \end{array}\right)$$

To determine the elastic coefficients, one can calculate the total energy for the unstrained system, *E*(*V*_0_,0), and for the strained solid, *E*(*V*,{*ε_i_*}), subject to various deformations:

(5) $$D=\left(\begin{array}{ccc} 1+\varepsilon_{1} & \varepsilon_{6}/2 & \varepsilon_{5}/2\\ \varepsilon_{6}/2 & 1+\varepsilon_{2} & \varepsilon_{4}/2\\ \varepsilon_{5}/2 & \varepsilon_{4}/2 & 1+\varepsilon_{3} \end{array}\right)$$

Accordingly, within the tetragonal-form effective model adopted for the clusters, the strain-energy density is written as:

(6) $$\frac{∆E}{V_{0}}=\frac{1}{2}C_{11}\left(\varepsilon_{1}^{2}+\varepsilon_{2}^{2}\right)+\frac{1}{2}C_{33}\varepsilon_{3}^{2}+C_{12}\varepsilon_{1}\varepsilon_{2}+C_{13}\left(\varepsilon_{1}\varepsilon_{3}+\varepsilon_{2}\varepsilon_{3}\right)+\frac{1}{2}C_{44}\left(\varepsilon_{4}^{2}+\varepsilon_{5}^{2}\right)+\frac{1}{2}C_{66}\varepsilon_{6}^{2}$$

Within the six-coefficient tetragonal-form model adopted here, six independent deformation modes are sufficient to determine the effective coefficients of the anatase TiO_2_ clusters. For instance, the set of six *Tetra-elastic* strain patterns [30,48] were designed for periodic tetragonal crystals and implemented within the WIEN2k framework, combining volume-conserving orthorhombic and monoclinic distortions with volume-changing deformations to access the independent elastic constants.

In the present work, we adopt a different set of deformation modes that probes the same elastic coefficients through energy–strain curvatures but can be applied directly to Cartesian atomic coordinates, making it more natural for finite clusters treated within ORCA [63]. Furthermore, to perform internal consistency checks we ran two additional strain patterns that provide redundant tests on the extracted elastic response.

All deformations were applied in a cluster-fixed Cartesian reference frame, with the *z*-axis aligned with the long axis of the bipyramidal clusters (approximately along the anatase [001] direction), and the *x*- and *y*-axes lying in the transverse plane (approximately along [100] and [010], respectively).

The choice of the 6 + 2 deformations is detailed below. They were chosen to probe distinct combinations of elastic coefficients and provide partial redundancy, allowing internal consistency checks of the extracted coefficients:

- ***D*_1_** is a biaxial strain in the basal plane, a deformation that corresponds to equal in-plane strains along *x* and *y*, probing the combined response associated with the sum of *C*_11_ and *C*_12_.
- ***D*_2_** is an orthorhombic basal-plane strain which introduces opposite deformations along *x* and *y* and isolates the elastic anisotropy within the basal plane, providing access to the difference *C*_11_ − *C*_12_.
- ***D*_3_** is a uniaxial strain along the long axis, probing the elastic response along the apical direction and is associated with the effective axial stiffness *C*_33_.
- ***D*_4_** is a coupled axial–transverse strain which combines strains along the basal plane and the long axis, allowing access to elastic cross terms involving *C*_13_ in combination with *C*_11_ and *C*_33_.
- ***D*_5_** is a shear strain in the *yz* plane, probing the resistance to shear involving the apical direction and corresponds to the elastic coefficient *C*_44_.
- ***D*_6_** is a shear strain in the *xy* plane, which explores in-plane shear and is associated with the elastic coefficient *C*_66_.
- ***D*_7_** is a strain along the *x* axis allowing for the redundant determination of *C*_11_.
- ***D*_8_** is an isotropic volumetric strain, which allows an analysis of the overall compressional response of the cluster and provides a consistency check on combinations of elastic coefficients obtained from the previous deformation modes.

(7) $$D_{1}=\left(\begin{array}{ccc} 1+\delta & 0 & 0\\ 0 & 1+\delta & 0\\ 0 & 0 & 1 \end{array}\right)\;\;\;D_{2}=\left(\begin{array}{ccc} 1+\delta & 0 & 0\\ 0 & 1-\delta & 0\\ 0 & 0 & 1 \end{array}\right)\;\;D_{3}=\left(\begin{array}{ccc} 1 & 0 & 0\\ 0 & 1 & 0\\ 0 & 0 & 1+\delta \end{array}\right)\;D_{4}=\left(\begin{array}{ccc} 1+\delta & 0 & 0\\ 0 & 1 & 0\\ 0 & 0 & 1+\delta \end{array}\right)\;\;\;D_{5}=\left(\begin{array}{ccc} 1 & 0 & 0\\ 0 & 1 & \gamma /2\\ 0 & \gamma /2 & 1 \end{array}\right)\;\;\;D_{6}=\left(\begin{array}{ccc} 1 & \gamma /2 & 0\\ \gamma /2 & 1 & 0\\ 0 & 0 & 1 \end{array}\right)\;D_{7}=\left(\begin{array}{ccc} 1+\delta & 0 & 0\\ 0 & 1 & 0\\ 0 & 0 & 1 \end{array}\right)\;\;\;D_{8}=\left(\begin{array}{ccc} 1+\delta & 0 & 0\\ 0 & 1+\delta & 0\\ 0 & 0 & 1+\delta \end{array}\right)$$

To assess departures from the tetragonal relations, additional *y*-uniaxial and *xz*-shear deformations were evaluated for the 2-layer-cut clusters *n* = 151, 70 and 21. These imposed ε*_yy_* = δ and ε*_xz_* = ε*_zx_* = *γ*/2, respectively, using the same optimized reference geometries, functional, numerical settings and strain amplitudes as the original calculations.

For each deformation mode, symmetric tensile and compressive strains *δ* = ±0.003 and ±0.006 were applied, whereas the shear deformations used *γ* = ±0.02 and ± 0.04. Each energy–strain fit used five configurations: the undeformed reference and four strained configurations. Deformations were applied affinely to all atomic coordinates, and the total energy was evaluated for each strained configuration without further atomic relaxation (clamped-ion approach). The resulting curvatures describe the total-energy response to the prescribed affine deformations, including electronic relaxation but excluding internal atomic relaxation under strain. In the harmonic regime, allowing internal relaxation while preserving the imposed deformation would lower or leave unchanged the energy curvature along that deformation path. Every coefficient reported below is therefore a clamped-ion effective coefficient. The redundant deformation checks in Section 3.2 test the internal consistency of the fitted curvatures within the adopted six-coefficient model; they do not test the omitted internal-relaxation contribution or establish exact tetragonal symmetry of the cluster response.

For an exactly stationary reference geometry, the first derivative of the energy with respect to strain vanishes. Nevertheless, the linear coefficient *q*_1_ was retained in every quadratic fit to detect residual slopes or asymmetry. The quadratic approximation was assessed using the fit quality, the standard error of the curvature, and the agreement between curvatures obtained from the inner and outer strain pairs (Table S1). Substituting the strain tensor corresponding to each of the eight deformation modes into the expansion of Equation (6) yields the specific energy–strain relations summarized in Equation (8), which link the curvature of each fitted parabola directly to the corresponding combination of elastic coefficients.

(8) $$\frac{∆E_{1}}{V_{0}}=\left(C_{11}+C_{12}\right)\delta^{2}\;\;\;\;\;\frac{∆E_{2}}{V_{0}}=\left(C_{11}-C_{12}\right)\delta^{2}\;\;\;\;\;\frac{∆E_{3}}{V_{0}}=\frac{1}{2}C_{33}\delta^{2}\;\frac{∆E_{4}}{V_{0}}=\frac{1}{2}\left(C_{11}+C_{33}+{2C}_{13}\right)\delta^{2}\;\;\;\;\;\frac{∆E_{5}}{V_{0}}=\frac{1}{2}C_{44}\gamma^{2}\;\;\;\;\;\frac{∆E_{6}}{V_{0}}=\frac{1}{2}C_{66}\gamma^{2}\;\frac{∆E_{7}}{V_{0}}=\frac{1}{2}C_{11}\delta^{2}\;\;\;\;\;\frac{∆E_{8}}{V_{0}}=\left(C_{11}+C_{12}+2C_{13}+\frac{1}{2}C_{33}\right)\delta^{2}$$

Conversion of the energy–strain curvatures into quantities with stiffness units requires a reference volume. The conventions used here and the conclusions that do or do not depend on that choice are defined in Section 2.3.

The effective bulk modulus (*B*) was obtained in two ways. First, it was determined directly from the isotropic deformation curve. Second, it was reconstructed from the fitted effective stiffness coefficients using the tetragonal Voigt expressions.

(9) $$B_{V}=\frac{1}{9}\left[2\left(C_{11}+C_{12}\right)+C_{33}+4C_{13}\right]$$

The effective shear modulus of the clusters was determined based on the Voigt relation:

(10) $$G_{V}=\frac{1}{15}\left(2C_{11}-C_{12}+C_{33}-2C_{13}+{6C}_{44}+3C_{66}\right)$$

In addition, the Reuss approach uses effective compliance constants (inverse matrix of the Voigt stiffness matrix), whereas the Hill approach is the average of the corresponding Voigt and Reuss values [23,64]. In turn, Hill’s effective coefficients are the basis for the calculation of effective Poisson’s ratio and Young’s effective modulus [23].

(11) $$E=\frac{9B_{H}G_{H}}{3B_{H}+G_{H}}$$

(12) $$\nu =\frac{3B_{H}-2G_{H}}{6B_{H}+{2G}_{H}}$$

The universal elastic anisotropy index was calculated as [65]:

(13) $$A^{U}=5\frac{G_{V}}{G_{R}}+\frac{B_{V}}{B_{R}}-6$$

### 2.3. Reference Volume Normalization and Comparative Analysis

For a periodic solid, elastic coefficients are defined as second derivatives of the energy density with respect to strain. For a finite cluster, the energy curvature remains well defined, but the volume required to convert that curvature into pressure units is not unique. For a chosen reference volume *V*_0_ = *V*_ref_, a volume-normalized stiffness coefficient is defined as:

(14) $$C_{\mathrm{ij}}^{\mathrm{ref}}=\frac{1}{V_{\mathrm{ref}}}{\left.\frac{\partial^{2}E}{\partial \varepsilon_{i}\partial \varepsilon_{j}}\right|}_{0}$$

Different plausible definitions of cluster volume can therefore produce different numerical elastic coefficients from exactly the same energy–strain data. More importantly, if the ratio between the reference volumes varies with cluster size or morphology, normalization can create, suppress, or reverse apparent size trends and structure–property correlations. The volume definition must therefore be treated as part of the reported quantity rather than as a purely technical detail.

This problem is particularly important for the present clusters. A convex hull constructed through the nuclear positions excludes the outer part of the volume associated with the surface atoms and consequently gives a small volume, especially for the smallest clusters. For the hydroxylated cut clusters, a hull including all atoms also incorporates the protruding hydroxyl groups and can substantially enlarge the assigned particle volume. Thus, neither geometric definition provides a neutral basis for comparing clusters that differ in size, shape, and passivation.

Three volume conventions were considered: the bulk-equivalent reference volume *V*_beq_ = *nv*_anat_, the TiO_2_-core volume *V*_core_, defined by the convex hull of the TiO_2_-framework atoms (including its surface atoms but excluding hydroxyl groups), and the total volume *V*_tot_, defined by the convex hull of all atoms, including hydroxyl passivation. Note that for the bipyramidal clusters *V*_core_ = *V*_tot_, because these clusters contain no hydroxyl passivation. Here, *v*_anat_ is the equilibrium volume per TiO_2_ formula unit in crystalline anatase, equal to 34.06 Å^3^ per TiO_2_ formula unit. The three reference volumes and their ratios for each cluster are listed in the Supplementary Material (Table S2).

The energy–strain calculation itself can be separated from this volume choice. For each deformation, the unnormalized energy differences were fitted as:

∆*E*(*δ*) = *q*_0_ + *q*_1_*δ* + *q*_2_*δ*^2^ (15)

The energy curvature at zero strain is 2*q*_2_, being independent of the reference volume convention. It is therefore the fundamental volume-independent output of the procedure. As 2*q*_2_ is an extensive quantity, 2*q*_2_/*n* is the intensive quantity that can provide the curvature per TiO_2_ unit comparable among clusters of different sizes. A reference volume enters only when the curvatures, or the combinations of curvatures associated with the applied deformation modes, are converted into effective elastic coefficients expressed in GPa. Standard errors of *q*_2_ were obtained from the least-squares covariance matrix.

When using the bulk-equivalent volume, the coefficient becomes:

(16) $$C_{\mathrm{ij}}^{\mathrm{beq}}=\frac{1}{v_{\mathrm{anat}}}\left(\frac{1}{n}{\left.\frac{\partial^{2}E}{\partial \varepsilon_{i}\partial \varepsilon_{j}}\right|}_{0}\right)$$

Thus, $C_{\mathrm{ij}}^{\mathrm{beq}}$ expresses the energy curvature per TiO_2_ unit using the same conversion factor, 1/*v*_anat_, for every cluster. It avoids introducing a geometrical scale factor that is itself correlated with cluster size, shape, or hydroxyl passivation. However, *V*_beq_ is not a physical external volume of the nanocluster. The resulting quantities are therefore termed bulk-equivalent stiffness coefficients and should not be interpreted as experimental particle moduli defined using a measured particle envelope. The two convex-hull conventions are retained to show how strongly the dimensional results depend on the assigned geometrical volume.

It is important to note that changing the volume normalization does not alter any calculated energy or energy curvature but may change whether a coefficient appears to increase or decrease and which coefficient appears closest to being invariant. Absolute coefficients, dimensional moduli, percentage differences from bulk, size trends, and correlations involving dimensional elastic quantities are therefore not generally invariant under a change in volume convention. If we apply a reference volume change to the same energy–strain data, say from “beq” to “core”, the corresponding coefficients are related by:

(17) $$C_{\mathrm{ij}}^{\mathrm{core}}=\frac{V_{\mathrm{beq}}}{V_{\mathrm{core}}}C_{\mathrm{ij}}^{\mathrm{beq}}$$

The dimensional Voigt, Reuss, and Hill bulk and shear moduli and Young’s modulus scale by the same factor *V*_beq_/*V*_core_. Quantities that are homogeneous of degree zero in the stiffness tensor are unchanged by this rescaling. These include ratios between coefficients of the same cluster, *B*/*G*, Poisson’s ratio, and the universal elastic anisotropy index. Rescaling by a positive factor also preserves the signs of the individual coefficients, as well as satisfaction of the mechanical-stability inequalities. In contrast, dimensional elastic quantities, including the absolute coefficient magnitudes and moduli, scale with the chosen reference volume.

To analyze morphology effects, we assessed the changes in coefficients of the cut clusters with respect to their bipyramidal parent. In this case, the role of the volume normalization convention is even more important, as the scaling factor, the ratio of the two reference volumes, generally differs among clusters. If the convention for the reference volume is changed, the ratio between the elastic coefficient of the cut clusters and their bipyramidal parents is:

(18) $$\frac{C_{\mathrm{ij}}^{\mathrm{core},\mathrm{cut}}}{C_{\mathrm{ij}}^{\mathrm{core},\mathrm{bip}}}=\lambda_{\mathrm{beq}\to \mathrm{core}}\frac{C_{\mathrm{ij}}^{\mathrm{beq},\mathrm{cut}}}{C_{\mathrm{ij}}^{\mathrm{beq},\mathrm{bip}}}$$

where

(19) $$\lambda_{\mathrm{beq}\to \mathrm{core}}=\frac{V_{\mathrm{beq},\mathrm{cut}}/V_{\mathrm{core},\mathrm{cut}}}{V_{\mathrm{beq},\mathrm{bip}}/V_{\mathrm{core},\mathrm{bip}}}$$

For a given parent–cut pair, *λ*_beq→core_ is a positive volume factor that is identical for all six elastic coefficients. Multiplication by the same positive λ preserves the ordering of the six coefficient ratios. Thus, if one coefficient has a larger cut-to-parent ratio than another under one convention, it retains the larger ratio under every convention. However, multiplication by λ does not preserve the distance of a ratio from unity. The sign and magnitude of the percentage change, and the identification of the coefficient closest to being invariant, can therefore depend on the volume convention.

To study size trends, we examined the elastic properties as functions of *n*^−1/3^. For geometrically similar particles, *n*^−1/3^ is proportional to the surface-to-volume ratio because particle volume scales as *n* and surface area as *n*^2/3^. However, we note that the present parent and cut clusters are not geometrically self-similar, and cutting also changes composition and hydroxyl termination. Moreover, all clusters remain strongly surface dominated and do not contain a clearly developed bulk-like core.

Parent–cut comparisons retain the advantage that every cut cluster is related to a specified bipyramidal parent. Nevertheless, cutting simultaneously changes Ti count, aspect ratio, exposed facets, coordination, stoichiometry, and hydroxyl passivation. The observed differences therefore describe the combined cutting-and-passivation response and cannot be assigned uniquely to morphology.

### 2.4. Computational Details

Density functional theory (DFT) [66] is now well established for predicting TiO_2_ properties and explaining experimental data, and despite known limitations [67] can treat molecular systems of several hundred atoms at moderate computational cost [24], well suited to the cluster sizes considered here, which range up to 165 Ti atoms.

All calculations were carried out within density functional theory using the ORCA [63,68] suite of programs, version 6.1.0. The exchange–correlation functional B3LYP [69,70] was used with the def2-SVP basis set [71]. Using ORCA with the def2-SVP basis set paired with the def2/J auxiliary set allows the calculation to exploit the RIJCOSX approximation, accelerating the evaluation of Coulomb and exchange integrals to enable efficient geometric screening of large TiO_2_ clusters. No dispersion correction was applied, since the structural stability of titanium dioxide is dominated by ionic and polar covalent interactions rather than van der Waals forces.

For a basis-set sensitivity assessment, the *n* = 10 cluster was reoptimized using B3LYP/def2-TZVP with TightSCF. The isotropic (*D*_8_), axial (*D*_3_), and basal-shear (*D*_6_) energy–strain curves were then evaluated using the same strain amplitudes and fitting procedure as for def2-SVP.

To counter the electronic convergence bottlenecks typical of transition metal systems, enforcing TightSCF thresholds alongside the SlowConv convergence strategy ensures robust, physically meaningful wavefunctions without sacrificing the performance gains achieved by the double-zeta framework. All clusters were treated as neutral with singlet multiplicity. Geometry optimizations used ORCA’s default geometry convergence criteria. Single-point energy calculations at strained geometries were carried out at the same level of theory.

### 2.5. Structural and Electronic Descriptors and Statistical Analysis

**Structural descriptors.** Five descriptors were computed for each cluster from the optimized, unstrained geometry. First, cluster size was represented by the number of Ti atoms, *n*, and by *n*^−1/3^, which provides a simple measure proportional to the inverse linear size for clusters of comparable atomic density. Second, the dimensions *L_x_*, *L_y_*, and *L_z_* were obtained from the extent of the optimized nuclear coordinates along the corresponding Cartesian directions, where the extrema were evaluated using TiO_2_-framework atoms. Third, the aspect ratio, *AR*, and the transverse geometrical asymmetry, *δ_xy_*, were derived from these dimensions: *AR* compares the axial dimension with the mean transverse dimension, while *δ_xy_* describes the departure from equality of the two transverse dimensions, defined as:

(20) $$\mathrm{AR}=\frac{L_{z}}{(L_{x}+L_{y})/2}\;\mathrm{and}\;\Delta_{\mathrm{xy}}=100\frac{\left|L_{x}-L_{y}\right|}{(L_{x}+L_{y})/2}$$

Fourth, the coordination number of each Ti atom was determined by counting all oxygen neighbors, including hydroxyl oxygen atoms, within a Ti–O distance cutoff of 2.70 Å; the number and fraction of Ti atoms with coordination number *k* were denoted *N_k_* and *f_k_* = *N_k_*/*n*, respectively. The selection and robustness of this cutoff are examined in Section 3.1 and Figure S1 of the Supplementary Material. Fifth, mean Ti–O distances and their standard deviations as well as the mean O–Ti–O angles and their standard deviations were calculated over the framework atoms; together these describe the average local geometry and the dispersion of bonding environments within each cluster.

**Electronic descriptors.** Four descriptors were derived from the electronic structure of each optimized cluster, computed at the same level of theory used for the structural calculations. First, for each Ti atom, all Ti–O Mayer bond orders involving that atom were averaged, after which the per-atom means were grouped by Ti coordination class *k* to obtain the class mean ⟨MBO⟩*_k_* and its standard deviation. Second, Hirshfeld charges on Ti atoms were grouped using the same coordination classification, with ⟨*Q*_Ti_⟩*_k_* denoting the mean charge of the Ti atoms in class *k*. Third, cluster-averaged charges (⟨*Q*_Ti_⟩ and ⟨*Q*_O_⟩, with their standard deviations) were calculated over all atoms. Fourth, the frontier-orbital gap was taken as the difference between the Kohn–Sham energies of the lowest unoccupied (LUMO) and highest occupied (HOMO) molecular orbitals.

**Statistical analysis.** Three complementary checks were applied to each correlation. First, linear relationships were evaluated using the Pearson correlation coefficient *r*, with two-tailed *p*-values. Second, Spearman’s rank coefficient *ρ* was calculated to determine whether the principal conclusions depended on the assumption of linearity. Third, sensitivity to individual clusters was evaluated by leave-one-out analysis: each correlation was recalculated after removing one cluster in turn, and the minimum and maximum resulting *r* values were recorded; a correlation was considered sign-stable when all leave-one-out values had the same sign. With nine clusters drawn from three parent families, and with each cut cluster paired to its parent, the observations are not independent and the analysis is exploratory: the coefficients describe the present set rather than support inference about anatase nanoparticles in general. Every correlation quoted below is therefore reported in Table S5 with its Pearson and Spearman coefficients, its leave-one-out range, its adjusted *q* value, and the coefficients obtained separately within the bipyramidal and the cut family.

**Multiple-comparison correction.** Three descriptor families were tested against the elastic and electronic targets, with the two-tailed *p* values adjusted within each family using the Benjamini–Hochberg procedure [72], which controls the false-discovery rate (adjusted values are denoted *q*). First, the nine structural descriptors (*n*^−1/3^, *f*_4_–*f*_6_, *AR*, ⟨*d*_Ti-O_⟩*_k_*, *σ*_Ti-O_, ⟨*θ*_O-Ti-O_⟩, and *σ_θ_*) were tested against each elastic target. Second, the same nine structural descriptors were tested against each electronic target. Third, the eleven electronic descriptors (the gap, ⟨*Q*_Ti_⟩, ⟨*Q*_O_⟩ and their dispersions, and the coordination-resolved ⟨MBO⟩*_k_* and ⟨*Q*⟩*_k_*) were tested against each elastic target. The elastic targets are the six coefficients, *B*_H_, *G*_H_, *E*, the Cauchy pressure, *ν*, *B*/*G*, *A*ᵁ, *C*_33_/*C*_11_, *C*_12_/*C*_11_ and *C*_66_/*C*_11_, each under the three volume conventions. Unadjusted *p* values are quoted in Section 3.4, as the purpose is to compare normalization conventions rather than to select descriptors; the corresponding q values are given in the Supplementary Material (Table S5).

After the quantum-chemical calculations and primary data analysis had been completed, generative artificial intelligence tools were used during manuscript preparation and revision for language editing and internal consistency checks across the manuscript, Supplementary Materials, and source data. These checks included recalculation of selected correlations between structural, electronic, and mechanical descriptors, and of expanded stiffness matrices. The authors reviewed all generated outputs and verified the reported results against the source data; they take full responsibility for the content of this publication.

## 3. Results

The results are presented in successive levels. The first section summarizes the optimized geometries of the nine clusters (*n* = 10–165 Ti atoms), through their coordination fractions *f*_4_, *f*_5_ and *f*_6_ and their aspect ratio *AR*, and the next validates the extraction of the effective elastic response. Section 3.3, Section 3.4 and Section 3.5 report the bulk-equivalent coefficients *C*_ij_^beq^ and effective Hill moduli *B*_H_, *G*_H_, *E*, *ν*, *B*/*G*, and *A*^U^, examine how much of the picture depends on the reference volume, and compare each cut cluster with its bipyramidal parent. The last two sections relate the structural and electronic descriptors to the elastic response.

### 3.1. Structural Characteristics of the Clusters

The calculations comprise nine optimized anatase-derived TiO_2_ nanoclusters, whose structures are shown in Figure 1. The set includes four bipyramidal clusters *n* = 10, 35, 84, and 165, three hydroxylated 2-layer-cut clusters *n* = 21, 70, and 151, and two hydroxylated 4-layer-cut clusters *n* = 40 and 121. The cut structures were generated from their corresponding bipyramidal parents, providing five parent–cut pairs for further comparative analysis. Table 1 summarizes the cluster compositions and morphologies together with the Ti coordination fractions (*f*_4_–*f*_6_), aspect ratio, *AR*, and transverse geometrical asymmetry, *δ_xy_*, as defined in Section 2.5. These coordination fractions reappear as explanatory variables later: Section 3.4 correlates the Hill bulk modulus with the fraction of 4-coordinate Ti, *f*_4_, and Section 3.7 examines coordination-resolved bonding and electronic structure in detail. The aspect ratios clearly distinguish the cluster morphologies, and reappear in Section 3.6 as the geometric variable linked to overall elastic anisotropy.

Ti coordination numbers, and the resulting *f*_4_–*f*_6_ classification in Table 1, were determined using a Ti–O distance cutoff of 2.70 Å, with every oxygen atom, including hydroxyl oxygen, counted on the same basis. The cutoff was selected from the complete Ti–O pairwise-distance distribution across the nine clusters (697 Ti atoms and 13,780 Ti–O pairs). The longest contact included in the first coordination shell is 2.674 Å, whereas the shortest excluded contact is 3.063 Å, leaving an empty interval between the two sets of contacts (Figure S1 of the Supplementary Material). Consequently, the coordination counts are unchanged for any cutoff from 2.70 to 3.00 Å. No Ti atom is 3-coordinate under this definition; the observed coordination classes are four, five, and six.

Hydroxyl oxygen atoms are not borderline cases. Across the five hydroxylated clusters, every terminal –OH oxygen lies 1.76–1.83 Å from its host Ti atom, while the next-nearest Ti atom is at least 3.63 Å away. Each hydroxyl oxygen is therefore unambiguously bonded to one Ti center and is included in that Ti atom’s coordination number, consistent with the role of hydroxylation in restoring the coordination of Ti at the cut surfaces.

The coordination fractions alone do not show where the most strongly altered Ti environments occur. The clearest examples are the four axial apex Ti atoms in the *n* = 35 and 84 bipyramids. Each is 4-coordinate according to the adopted definition but has a distinctly 3 + 1 local geometry: three short Ti–O bonds of approximately 1.59–1.90 Å and one substantially longer contact of 2.604–2.674 Å directed inward, almost parallel to the long axis of the bipyramid. The next-nearest oxygen is more than 4.2 Å away. These symmetry-related sites therefore differ markedly from 4-coordinate Ti environments containing four bonds of comparable length. Additional Ti atoms with elongated bonds form near-apical rings in the *n* = 35, 84, and 165 bipyramids. Thus, the most distinctive longer-bond environments in the parent clusters are concentrated at the apices and in the immediately adjacent layers.

Alignment of every cut cluster with its bipyramidal parent shows that the atoms removed by cutting form symmetric apical slabs. The axial apex sites and the surrounding subapical Ti atoms with altered local environments belong to these removed regions. Cutting also causes retained Ti atoms adjacent to the new surfaces to lose one, and in some cases two, framework oxygen neighbors. Hydroxylation restores part of this lost coordination, but the added Ti–OH bonds do not reproduce the orientation and connectivity of the removed Ti–O–Ti framework. Most of these changes are concentrated in the newly exposed terminal layers, located at approximately |*z*| = 12.0 and 7.2 Å in the two cuts of the *n* = 165 parent, |*z*| = 7.1 and 2.4 Å in the two cuts of the *n* = 84 parent, and |*z*| = 2.4 Å in the *n* = 35→21 pair.

The smaller clusters also exhibit secondary changes away from the immediate cut plane. In the 84→70 pair, altered environments occur at several lateral-edge Ti atoms, whereas in the 35→21 pair they extend to several equatorial or central sites. A further group of Ti atoms with unusually long, but excluded, Ti···O contacts occurs mainly near the outer equatorial region of the 165→151→121 family and in the *n* = 70 cluster. These contacts do not change the accepted coordination counts, but they reveal local distortion outside the apical region. The altered environments are therefore spatially organized into axial apical and subapical motifs, newly exposed cut-surface sites, and lateral or equatorial distortions. This distinction provides the structural basis for examining how cutting preferentially changes the axial response relative to the in-plane network in Section 3.5 and Section 4.2.

The coordination fractions depend strongly on cluster size. Within the bipyramidal series, *f*_6_ decreases from 0.430 at *n* = 165 to zero at *n* = 10, whereas *f*_4_ increases from 0.055 to 0.800; 5-coordinate Ti predominates at the intermediate sizes. No Ti atom has a coordination number below four under the adopted definition. Cutting modifies the coordination distribution, but not by producing a uniform change in every fraction. Four of the five cut clusters have a larger *f*_4_ than their bipyramidal parent; the exception is the 165→151 pair, for which *f*_4_ decreases slightly. The largest redistributions occur for the deeper cuts and the smaller parents, particularly for 84→40 and 35→21, whereas the shallower cuts produce smaller changes because hydroxylation restores part of the coordination lost at the newly exposed surfaces. The coordination fractions therefore describe the combined consequences of layer removal, structural relaxation, and hydroxyl passivation rather than the effect of cutting alone.

The aspect ratios for the bipyramidal clusters are *AR* = 2.06–2.76, reflecting their elongation along *z*. The 2-layer-cut structures span *AR* = 0.78–1.58, while the 4-layer-cut structures have *AR* = 0.55–0.99. Apical-layer removal therefore primarily reduces *L_z_* and transforms the elongated parent clusters into more compact geometries. The transverse geometrical asymmetry remains at or below 2.56% for six clusters and reaches a mismatch of 6.63%, 8.60%, and 9.64% for the *n* = 10, 40, and 21 clusters, respectively.

### 3.2. Energy–Strain Fits and Validation of the Effective Elastic Coefficients

**Figure 2:**
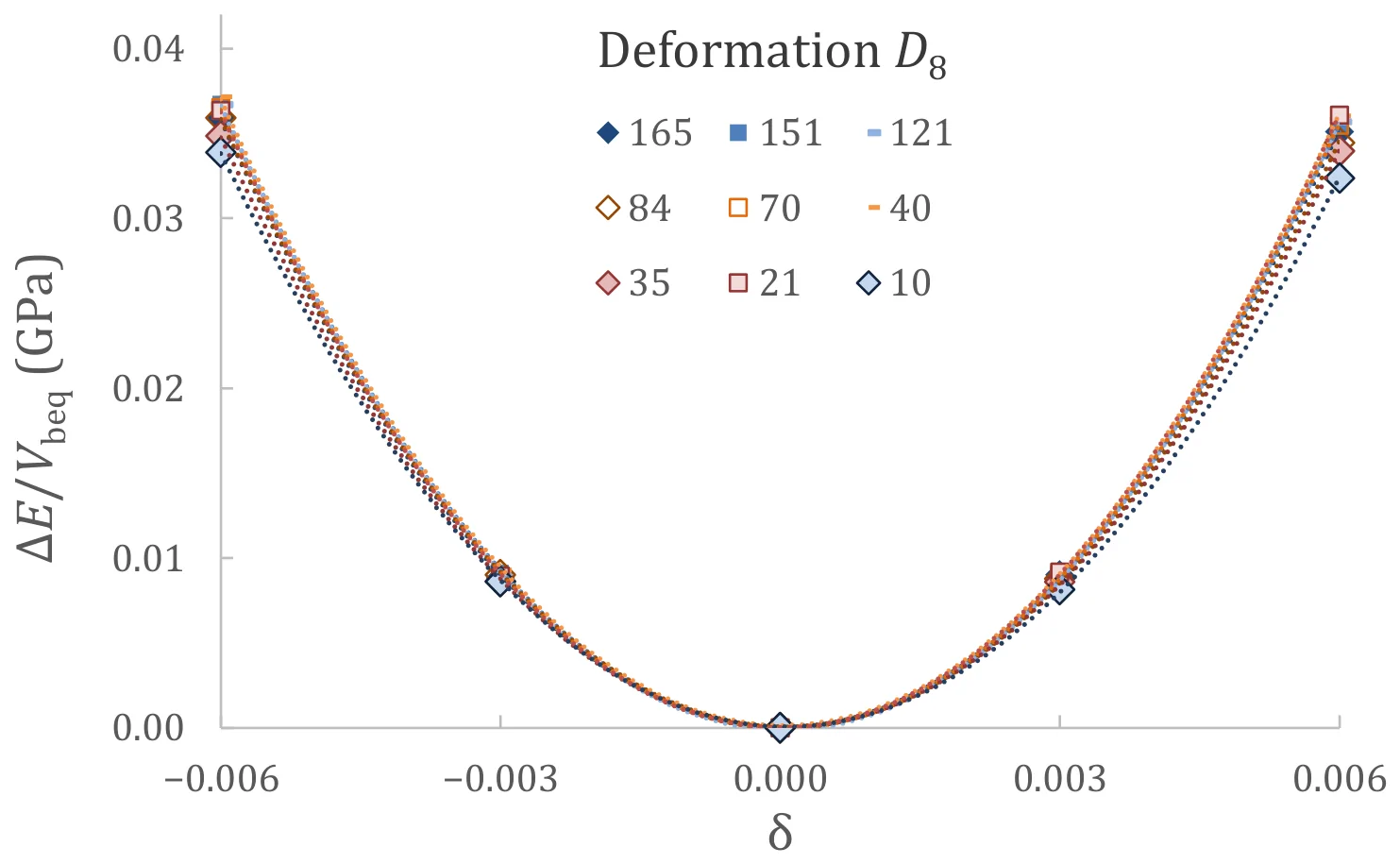
Bulk-equivalent strain-energy density, Δ*E*/*V*_beq_, as a function of isotropic strain *δ* for deformation *D*_8_ and all nine anatase-derived TiO_2_ nanoclusters. Symbols represent the calculated values relative to the unstrained structures, and dotted curves are least-squares quadratic fits. Diamonds, squares, and rectangles denote bipyramidal, 2-layer-cut, and 4-layer-cut clusters, respectively; the labels give the number of Ti atoms, *n*. All fits yield *R*^2^ ≥ 0.998; the fitted curvatures, their standard errors and R^2^ for all deformation modes are given in the Supplementary Material (Table S1).

For each cluster, the energy changes produced by the eight homogeneous deformation modes were fitted as quadratic functions of strain, as described in Section 2.2. Figure 2 shows the results for the isotropic deformation *D*_8_; the corresponding energy–strain curves for *D*_1_–*D*_7_ are provided in the Supplementary Material (Figure S2). All fits gave *R*^2^ ≥ 0.998 over the applied strain interval, supporting the quadratic representation used to determine the effective elastic coefficients. As *R*^2^ is insensitive to small departures from a parabola, the calculated standard error of each fitted curvature is below 3% (median 0.7%).

Two internal consistency checks compared effective elastic quantities determined through different deformation routes. First, *C*_11,calc_^beq^ was determined together with *C*_12_^beq^ from the two in-plane deformations *D*_1_ and *D*_2_, and compared with *C*_11,D7_^beq^, obtained independently from the uniaxial deformation *D*_7_ using Equation (8). Second, *B*_V,calc_^beq^, calculated from the six reported elastic coefficients using Equation (9), was compared with *B*_V,*D*8_^beq^, determined directly from the isotropic deformation *D*_8_. The results are given in Table 2.

The two bulk-modulus determinations agree within 3.98% for all nine clusters. For the four bipyramidal clusters, the two determinations of *C*_11_^beq^ agree within 4.03%. The two routes probe the same coefficients through different deformations, so their agreement supports the internal consistency of the fitting procedure. It does not test the accuracy of the coefficients themselves, which rests on the level of theory and on the clamped-ion treatment.

The additional deformation tests quantified departures from the transverse equalities assumed in the tetragonal-form model (Tables S7–S10). The reconstructed differences between *C*_11_ and *C*_22_, normalized by their mean, were approximately 0.9%, 1.5% and 19.1% for *n* = 151, 70 and 21, respectively; *C*_55_ differed from *C*_44_ by approximately 0.1%, −1.7% and −11.7%. Combining the additional curves with the existing normal-strain deformations allowed reconstruction of the normal-strain block without imposing *C*_11_ = *C*_22_ or *C*_13_ = *C*_23_. The reconstruction equations and expressions used to calculate the aggregate elastic quantities are provided in the Supplementary Material, Equations (S1)–(S7). With the three shear stiffnesses treated separately and unmeasured couplings retained at zero, the Hill moduli changed by less than 1% for *n* = 151 and 70, and by 3.7% in bulk and −4.3% in shear for *n* = 21. The absolute point-estimate change in Aᵁ was below 0.01 in each case. These tests support the use of the compact model for the assessed aggregate quantities, while identifying appreciable component-level asymmetry in the smallest tested cut cluster. They do not establish that unmeasured couplings vanish or validate the untested 4-layer-cut series.

For *n* = 10, changing from the separately optimized B3LYP/def2-SVP structure to the B3LYP/def2-TZVP structure decreased the fitted isotropic curvature by 3.84% and *C*_33_ by 3.56%, while increasing *C*_66_ by 1.21% (Tables S11 and S12). These changes were comparable to the fitting and strain-window sensitivity. The comparison assesses the combined effects of basis enlargement and geometry reoptimization for the three sampled deformation modes.

### 3.3. Bulk-Equivalent Effective Elastic Coefficients and Moduli

**Figure 3:**
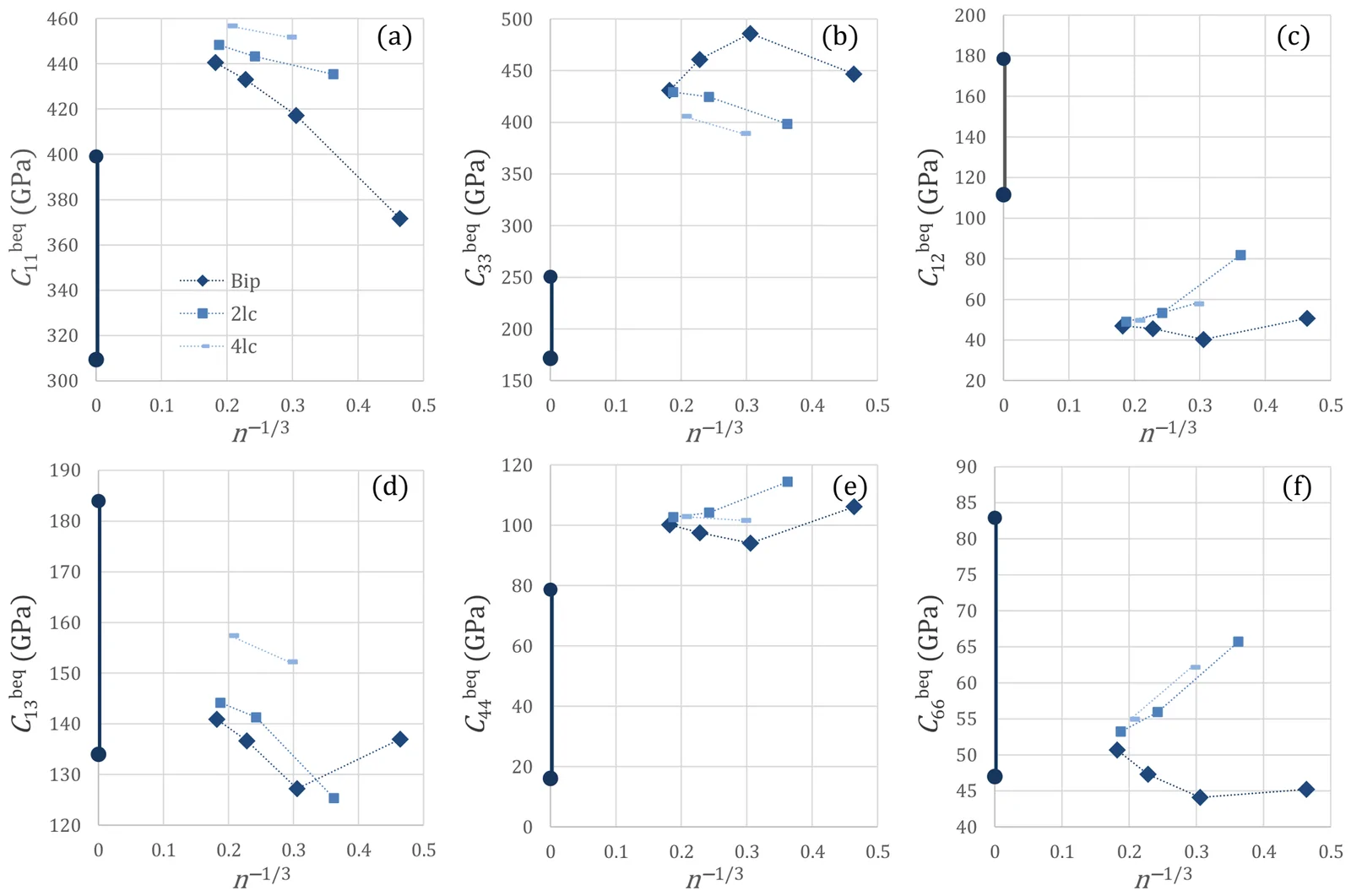
Bulk-equivalent effective elastic coefficients of the anatase-derived TiO_2_ nanoclusters as a function of inverse cluster size *n*^−1/3^. The in-plane direct stiffness coefficient *C*_11_^beq^ (**a**) axial direct stiffness coefficient *C*_33_^beq^ (**b**), in-plane coupling coefficient *C*_12_^beq^ (**c**), axial–basal coupling coefficient *C*_13_^beq^ (**d**), and shear coefficients *C*_44_^beq^ (**e**) and *C*_66_^beq^ (**f**) were obtained using *V*_beq_. Diamonds, squares, and rectangles denote bipyramidal, 2-layer-cut, and 4-layer-cut clusters, respectively. Vertical bars at *n*^−1/3^→0 span the range of published DFT values for crystalline anatase (Refs. [23,26,27,28,29,30,31,32,33,34,35]). Dotted lines connect clusters of the same morphology as guides to the eye.

Table 3 reports the six bulk-equivalent effective elastic coefficients of the nine clusters, while Figure 3 shows their variation with inverse cluster size, *n*^−1/3^, for the three morphological series. The range spanned by published DFT values for crystalline anatase (Refs. [23,26,27,28,29,30,31,32,33,34,35]) is shown at *n*^−1/3^→0 for reference.

Published DFT coefficients for crystalline anatase scatter widely with the functional and the method, so the clusters are compared with the range spanned by sixteen tensors (Refs. [23,26,27,28,29,30,31,32,33,34,35]; Table 3) and only statements that hold against every one of them are made. The cluster coefficients are clamped-ion, whereas the published bulk values were obtained using varied computational methods and, where stated, generally include ionic relaxation. The comparison therefore places the cluster response within the spread of published bulk results but does not isolate finite-size or morphology effects. Three components stand clearly outside the bulk range in every cluster: the axial direct stiffness *C*_33_^beq^ and the axial shear coefficient *C*_44_^beq^ exceed the largest published value, while the in-plane transverse coupling *C*_12_^beq^ falls below the smallest. The in-plane direct stiffness *C*_11_^beq^ exceeds the largest published value for eight clusters and lies within the range for *n* = 10. The remaining two coefficients stay mostly inside it: *C*_13_^beq^ falls below the range only for *n* = 35 and 21, and *C*_66_^beq^ only for *n* = 35 and 10. The *C*_13_^beq^ comparison for *n* = 21 is sensitive to the imposed transverse equality: reconstruction of the normal-strain block gives approximately 146 GPa under bulk-equivalent normalization, within the published bulk range. What distinguishes the clusters from the crystal is therefore not a uniform stiffening but a change in the shape of the tensor, concentrated in the axial and transverse-coupling components.

The size dependence of the bulk-equivalent coefficients is specific to both the tensor component and the morphological series (Figure 3): bulk-equivalent normalization does not produce a universal increase in the effective elastic coefficients as cluster size decreases. The in-plane direct stiffness *C*_11_^beq^ decreases as *n* decreases within all three morphological series. In the cut families, the in-plane coupling and shear coefficients *C*_12_^beq^ and *C*_66_^beq^ increase toward smaller clusters, whereas the axial direct stiffness *C*_33_^beq^ decreases. Within the bipyramidal series, the axial stiffness increases from *n* = 165 to 35 and then decreases at *n* = 10, while the remaining coupling and shear coefficients show weaker or nonmonotonic variations.

The bulk-equivalent effective Hill moduli derived from the reported coefficients, together with the three normalization-invariant elastic measures, are listed in Table 4. The effective coefficients and moduli under the other two volume conventions are given in the Supplementary Material (Table S3). In contrast to Figure 3, in Figure S3 we illustrate the importance of the volume normalization, plotting the dependence of the effective elastic coefficients obtained with the core hull convention on inverse cluster size *n*^−1/3^.

Compared with the published bulk range, the cluster moduli diverge more sharply than the bulk-equivalent coefficients themselves: the bulk modulus overlaps the bulk range, but the shear and Young’s moduli sit entirely above it. The effective Hill bulk modulus *B*_H_^beq^ spans a narrow range, which overlaps the upper part of the published bulk range, four clusters lying above its maximum. The shear and Young’s moduli separate more clearly: *G*_H_^beq^ and *E*^beq^ lie above the entire bulk ranges. Among the convention-free measures, Poisson’s ratio *ν* and the Pugh ratio *B*_H_/*G*_H_ fall below the bulk ranges, whereas the universal anisotropy index *A*^U^ lies inside the bulk range. The universal elastic anisotropy index also shows the broadest relative variation across the cluster set, which is why it serves below as the anisotropy measure.

### 3.4. Dependence of the Effective Elastic Properties on the Reference-Volume Convention

**Figure 4:**
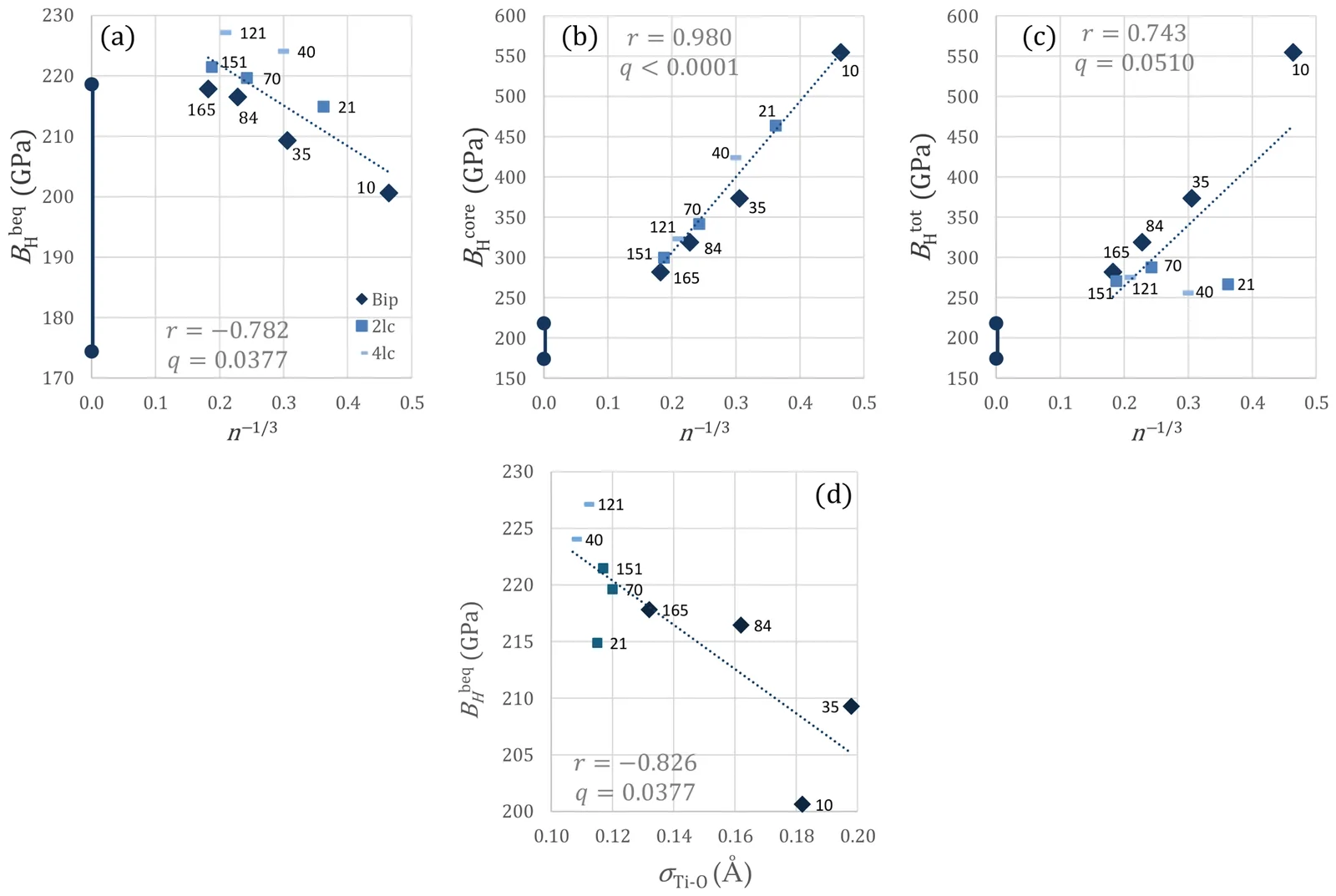
Size and structural dependence of the effective Hill bulk modulus of anatase-derived TiO_2_ nanoclusters. The moduli calculated using (**a**) the bulk-equivalent volume, *B*_H_^beq^, (**b**) the core volume, *B*_H_^core^, and (**c**) the total volume, *B*_H_^tot^, are plotted against inverse cluster size, *n*^−1/3^. (**d**) *B*_H_^beq^, versus the standard deviation of the Ti–O bond lengths, *σ*_Ti-O_. Diamonds, squares, and rectangles identify the bipyramidal, 2-layer-cut, and 4-layer-cut clusters, respectively. In panels (**a**–**c**), the vertical bars at *n*^−1/3^ = 0 span the Hill bulk modulus of bulk anatase, 174.4–218.6 GPa, obtained here from the sixteen published tensors of Refs. [23,26,27,28,29,30,31,32,33,34,35] by the same Voigt–Reuss–Hill procedure. Dotted lines show linear least-squares fits across the nine clusters, labeled by the number of Ti atoms, *n*. The reported *r* values are Pearson correlation coefficients; *q* values are the corresponding two-sided *p* values adjusted by the Benjamini–Hochberg procedure within the predefined family of structural descriptors tested against each modulus.

To determine how the reference-volume convention affects comparisons across the cluster set, the effective Hill bulk modulus was recalculated using *V*_beq_, *V*_core_, and *V*_tot_. The three results are derived from the same energy–strain curvatures and differ only in the volume used to express the response in pressure units. Table 5 summarizes the resulting modulus ranges and correlations with inverse cluster size and 4-coordinate Ti fraction, whereas Figure 4 displays the corresponding size trends.

Under bulk-equivalent normalization, the effective Hill bulk modulus spans 200.6–227.1 GPa and falls as the clusters get smaller (*r* = −0.782, *p* = 0.0128). This Pearson association remains below the Benjamini–Hochberg-adjusted threshold (*q* = 0.0377), although the weaker rank coefficient (*ρ* = −0.633, Spearman *p* = 0.067) provides less support for a monotonic relationship. The direction is nevertheless stable in the leave-one-out analysis, for which *r* ranges between −0.86 and −0.48. The other two moduli, *G*_H_^beq^ and *E*_beq_, show the same general behavior.

The four bipyramidal clusters provide a natural control series. They span the full investigated size range while sharing the same morphology and stoichiometric composition. Within this series, *B*_H_^beq^ decreases almost linearly with *n*^−1/3^ (*r* = −0.992, *R*^2^ = 0.984; Table S5). In contrast, the total energy per TiO_2_ unit increases linearly with the same size variable (*R*^2^ = 0.9937; Figure S4), as expected from the increasing surface contribution. The cluster set therefore addresses size effects clearly. What it does not show is surface stiffening: the smaller bipyramidal clusters have higher energies per TiO_2_ unit but lower bulk-equivalent moduli.

The root-mean-square residual from the *B*_H_^beq^ size trend is 1.2 GPa within the bipyramidal series and 5.3 GPa across all nine clusters. The larger scatter arises because the cut clusters are displaced from the bipyramidal trend. Size therefore controls the baseline response, while cutting introduces an additional morphology-dependent contribution examined in Section 3.5.

Beyond the size dependence, the bulk-equivalent response is also associated with local bond-length heterogeneity, decreasing as the Ti–O bond-length dispersion increases (*r* = −0.826, *p* = 0.0061, *q* = 0.0377; Figure 4d). This relationship is also supported by the rank correlation (*ρ* = −0.800, Spearman *p* = 0.0096), and its direction remains stable throughout the leave-one-out analysis (*r* = −0.883 to −0.803). Thus, within the investigated cluster set, a broader distribution of local Ti–O environments accompanies a softer bulk-equivalent response.

In contrast, core-hull normalization produces a much broader range of 281.9–554.7 GPa and a strong statistically significant positive correlation with *n*^−1/3^ (*r* = 0.980, *ρ* = 0.967, and *p* < 0.0001), which holds within each family as well (see Figure S3 in the Supplementary Material). Total-hull normalization gives 255.7–554.7 GPa and a positive Pearson coefficient (*r* = 0.743, *p* = 0.0219), but the relationship is not monotonic: the rank coefficient is *ρ* = 0.250 (*p* = 0.52), and within the cut clusters alone it reverses sign (*r* = −0.43). The apparent total-hull trend therefore mostly separates the bipyramids from the cut clusters instead of describing a size dependence.

Both hull conventions also push every cluster above the bulk range of 174.4–218.6 GPa obtained by the same Voigt–Reuss–Hill procedure, and the margin widens as the clusters shrink: the smallest reaches 554.7 GPa, roughly two and a half times the largest value reported for bulk anatase. The cause is arithmetic rather than physical. At fixed curvature a modulus scales as the reciprocal of the assigned volume, and the core hull contracts faster than the cluster itself, *V*_core_/*V*_beq_ falling from 0.773 at *n* = 165 to 0.362 at *n* = 10 (Table S2). What the hull conventions record is that contraction of the assigned volume, not a stiffening of the Ti–O network.

The correlation with the fraction of 4-coordinate Ti, *f*_4_, changes in the same way. It is negative for *B*_H_^beq^ (*r* = −0.778) and positive for *B*_H_^core^ (*r* = 0.949) and *B*_H_^tot^ (*r* = 0.765; Table 5). The bulk-equivalent and core-hull Pearson correlations remain significant after Benjamini–Hochberg adjustment (*q* = 0.0377 and 0.0003, respectively), whereas the total-hull correlation narrowly does not (*q* = 0.0510; Table S5). The rank correlation supports the core-hull relationship (*ρ* = 0.967) but is weaker under bulk-equivalent and total-hull normalization (*ρ* = −0.567 and 0.167, respectively). Because the underlying energy–strain curvatures are identical, the change in sign arises from the cluster-dependent ratios between the assigned reference volumes, as anticipated in Section 2.3. The apparent relationship between *f*_4_ and stiffness is therefore normalization-dependent.

### 3.5. Effective Elastic Response to Apical-Layer Removal

**Figure 5:**
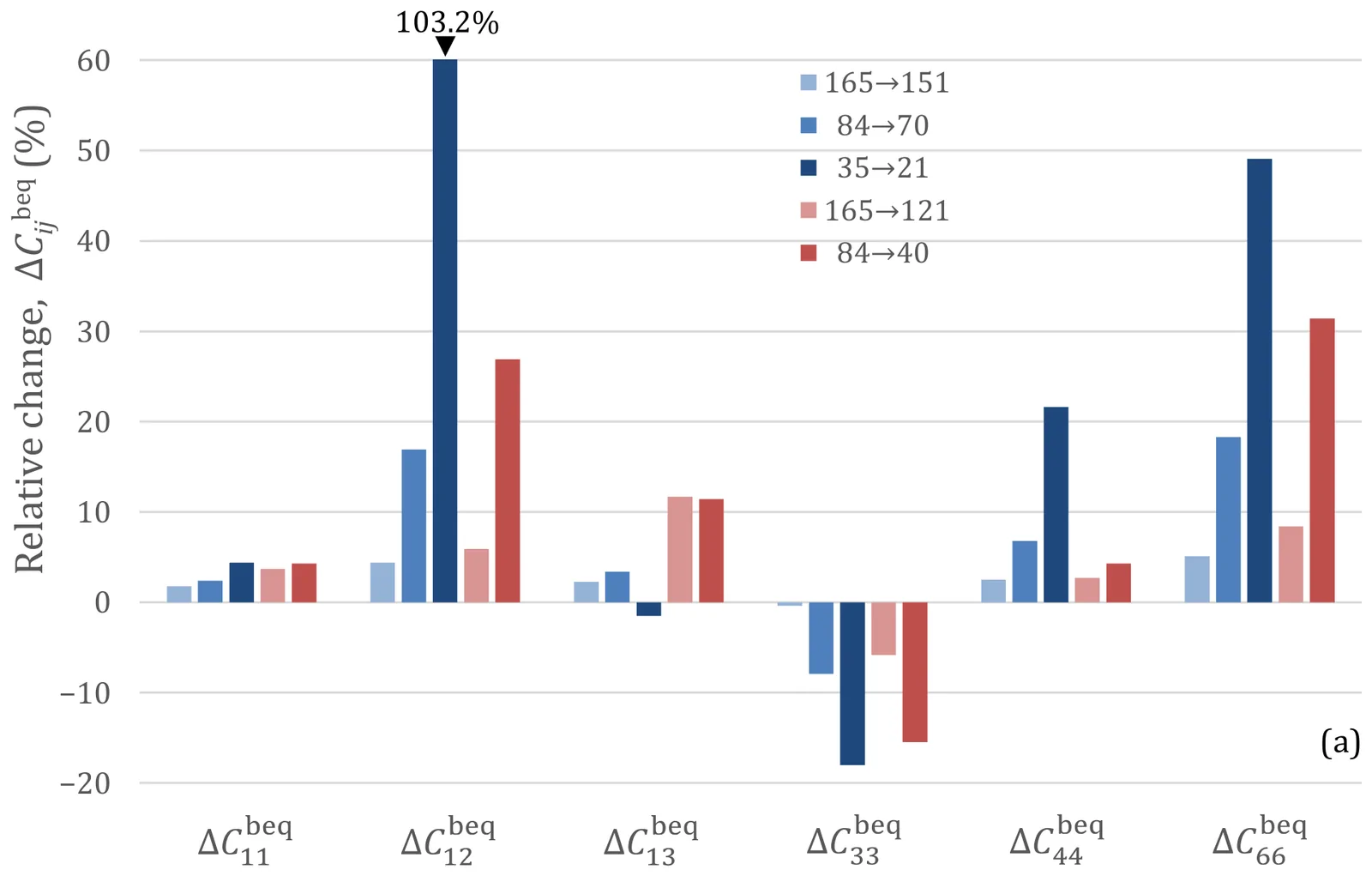

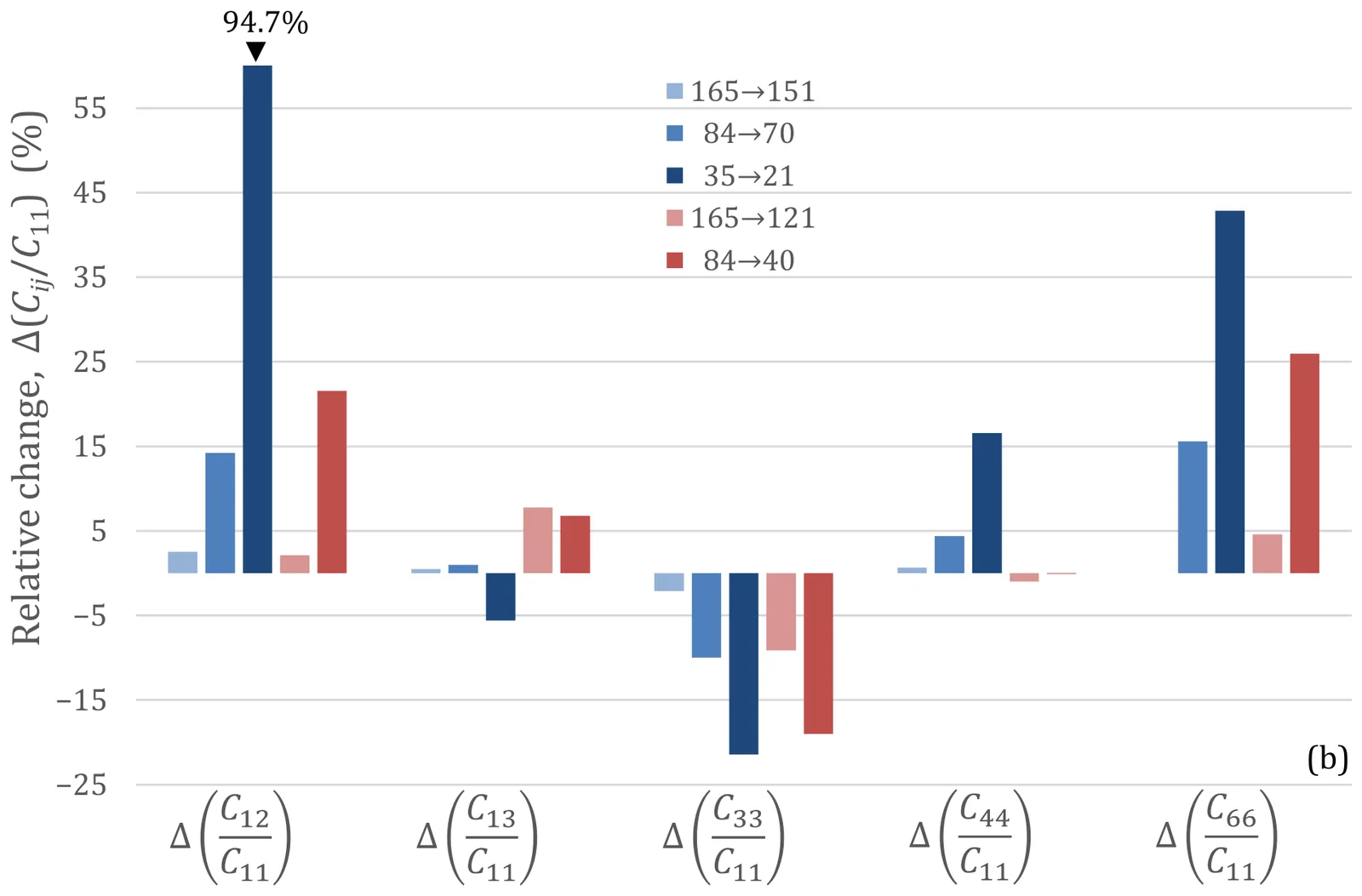
Changes on removing apical layers, for the five paired parent–cut comparisons: (**a**) relative changes in the bulk-equivalent effective elastic coefficients, Δ*C_ij_*^beq^; (**b**) relative changes in the five ratios of those coefficients to C_11_. Blue and red bars represent 2-layer and 4-layer cuts, respectively, and each legend entry identifies the number of Ti atoms in the parent and cut clusters. Positive values indicate an increase relative to the corresponding bipyramidal parent. The coefficients in panel (**a**) were obtained using *V*_beq_ = *n v*_anat_ and so depend on that choice; the ratios in panel (**b**) are unaffected by it, since changing the reference volume rescales every coefficient of a given cluster by the same factor. In each panel the largest C_12_ bar for the 35→21 pair, 103.2% in (**a**) and 94.7% in (**b**), is displayed above a truncated bar to preserve the scale of the remaining data.

The changes associated with removing apical atomic layers from the bipyramidal clusters and hydroxylating the resulting structures are quantified by the five paired comparisons. Figure 5 reports the relative changes in the bulk-equivalent effective elastic coefficients with respect to the corresponding bipyramidal structure. Removal of two atomic layers gives the comparisons 165→151, 84→70, and 35→21, while removal of four layers gives 165→121 and 84→40.

Consistent with their displacement from the bipyramidal size trend, *B*_H_^beq^ increases modestly in every parent–cut comparison. The increases are 1.7% and 4.3% for the 2- and 4-layer cuts of *n* = 165, 1.5% and 3.5% for the corresponding cuts of *n* = 84, and 2.7% for the 2-layer cut of *n* = 35. Cutting therefore produces a small increase in the aggregate modulus on top of the negative size trend. The much larger changes in selected tensor components show that this aggregate measure hides a strongly directional redistribution of stiffness.

Removing the apical layers changes every coefficient, but neither by the same amount nor always in the same direction. In the bulk-equivalent convention (Figure 5a), the in-plane direct stiffness *C*_11_ rises modestly in all five pairs (1.79–4.35%), whereas the in-plane transverse coupling *C*_12_ and the in-plane shear *C*_66_ rise far more (4.36–103.20% and 5.06–49.07%) and the axial shear *C*_44_ by 2.47–21.63%. The axial–in-plane coupling *C*_13_ grows in four pairs (2.30–11.74%) and falls by 1.48% for 35→21. Only the axial direct stiffness *C*_33_ decreases throughout, by 0.38–18.04%.

These individual signs depend on the volume convention, as Section 2.3 makes explicit. The ratios *C_ij_*/*C*_11_, however, are convention-independent because every coefficient of a given cluster is multiplied by the same normalization factor. In every parent–cut pair, *C*_33_/*C*_11_ decreases, whereas *C*_12_/*C*_11_ and *C*_66_/*C*_11_ increase. The axial-to-in-plane stiffness ratio *C*_33_/*C*_11_ falls in all five comparisons, from 2.1% for 165→151 to 21.5% for 35→21, whereas the transverse-coupling ratio *C*_12_/*C*_11_ and the in-plane shear ratio *C*_66_/*C*_11_ rise in all five (2.1–94.7% and 3.2–42.9%). The two remaining ratios, *C*_13_/*C*_11_ and *C*_44_/*C*_11_, take both positive and negative values across the five pairs. Cutting the apices thus does not simply stiffen or soften a cluster; it moves stiffness out of the axial direction and into in-plane coupling and shear (Table 4, Figure 5b).

How far the balance shifts depends on how much material is removed and how small the parent is. Among the two-layer cuts, the drop in *C*_33_/*C*_11_ grows steadily with decreasing parent size, from 2.1% (165→151) to 10.0% (84→70) and 21.5% (35→21). Cutting four layers instead of two deepens it further in both families where both cuts were made (9.1% for 165→121 and 19.0% for 84→40). The in-plane shear ratio *C*_66_/*C*_11_ behaves the same way, and so does *C*_12_/*C*_11_, with one exception: the four-layer cut of the *n* = 165 parent gives a slightly smaller increase than the two-layer cut (2.1% against 2.5%).

The smallest pair is the most extreme: for 35→21 the bulk-equivalent transverse coupling *C*_12_^beq^ rises by 103.2% and the ratio *C*_12_/*C*_11_ nearly doubles (94.7%), while the axial-to-in-plane ratio falls by more than a fifth. Both increases should be read against their small starting value, since *C*_12_ is the smallest of the six coefficients in the parent cluster. These shifts accompany the loss of elongation and the growth of under-coordinated Ti sites listed in Table 1, a connection taken up in Section 3.6.

### 3.6. Shape and Local-Distortion Relationships with the Effective Elastic Response

**Figure 6:**
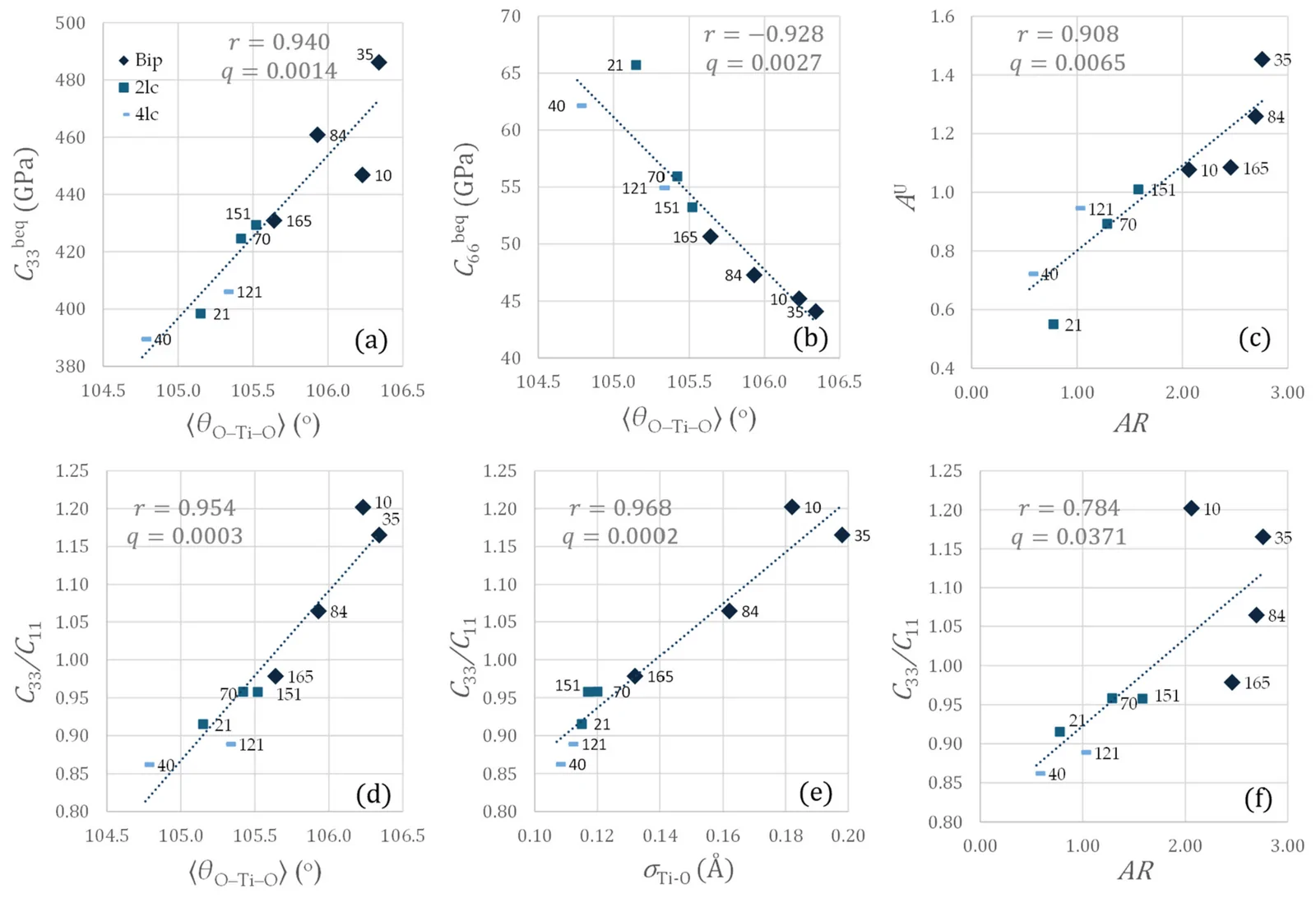
Correlations between structural descriptors and the effective elastic response of anatase-derived TiO_2_ nanoclusters: (**a**) *C*_33_^beq^ and (**b**) *C*_66_^beq^ versus the mean O–Ti–O bond angle, ⟨*θ*_O−Ti−O_⟩; (**c**) the universal elastic anisotropy index, *A*^U^ versus aspect ratio *AR*; and *C*_33_/*C*_11_ versus (**d**) ⟨*θ*_O−Ti−O_⟩; (**e**) the standard deviation of the Ti–O bond lengths, σ_Ti-O_, and (**f**) *AR*. Diamonds, squares, and rectangles identify the bipyramidal, 2-layer-cut, and 4-layer-cut clusters, respectively. Dotted lines show linear least-squares fits across the nine clusters, which are labeled by the number of Ti atoms, *n*. The reported *r* values are Pearson correlation coefficients; *q* values are the corresponding two-sided *p* values adjusted by the Benjamini–Hochberg procedure within the predefined family of structural descriptors tested against each elastic target.

To examine how cluster shape and local distortion relate to the directional elastic response, the aspect ratio *AR*, mean O–Ti–O angle, ⟨θ_O-Ti-O_⟩, and Ti–O bond-length dispersion, *σ*_Ti–O_, were compared with the bulk-equivalent elastic coefficients and with the normalization-independent measures *A*^U^ and *C*_33_/*C*_11_. Figure 6 presents six relationships that remain significant after Benjamini–Hochberg adjustment. Panels (a) and (b) relate the mean bond angle to the opposing responses of *C*_33_^beq^ and *C*_66_^beq^, while panels (c)–(f) connect cluster shape and local structural distortion with elastic anisotropy. Two additional significant correlations of *A*^U^, with ⟨θ_O-Ti-O_⟩, (*q* = 0.0140) and with *σ*_Ti–O_, (*q* = 0.0273), are reported in the Supplementary Material (Table S5).

The mean O–Ti–O angle is associated with opposite changes in the axial direct and in-plane shear responses. *C*_33_^beq^ increases with ⟨θ_O-Ti-O_⟩, whereas *C*_66_^beq^ decreases. Thus, larger mean angles accompany greater axial direct stiffness but weaker in-plane shear stiffness. The opposing signs show that the structural variation produces a directional redistribution of stiffness rather than a uniform change in all elastic coefficients.

Cluster aspect ratio is positively correlated with both measures of elastic anisotropy. Its relationship with the universal anisotropy index is strong and statistically significant (Figure 6c), with the elongated bipyramidal clusters occupying the high-*AR*, high-*A*^U^ region and the more compact cut clusters occupying lower values. The relationship also holds within each morphology (*r* = 0.810 for the bipyramids and *r* = 0.767 for the cut clusters), indicating that it is not merely a contrast between elongated and compact morphologies.

The axial-to-in-plane stiffness ratio likewise increases with aspect ratio (Figure 6f). Within the three parent families, the universal anisotropy index decreases from 1.085 to 1.010 and 0.946 for the *n* = 165 family, from 1.259 to 0.892 and 0.722 for the *n* = 84 family, and from 1.453 to 0.550 for the *n* = 35 family as the clusters are progressively cut.

These changes associate the loss of cluster elongation with a more balanced axial and in-plane elastic response. The *C*_33_/*C*_11_ relationship, however, is influenced by differences between the morphology groups, *r* = −0.311 within the four bipyramids but *r* = 0.882 within the cut clusters (Table S5). The local structural descriptors considered below provide more consistent correlates of this ratio.

Local geometric descriptors show still stronger and more morphology-consistent relationships with the axial-to-in-plane stiffness ratio. The mean O–Ti–O angle is positively correlated with *C*_33_/*C*_11_ (Figure 6d), with the same direction within the bipyramidal and cut-cluster subsets (*r* = 0.957 and 0.861, respectively). Ti–O bond-length dispersion gives the strongest relationship (Figure 6e), which also persists within both subsets (*r* = 0.923 and 0.969). These associations are therefore not produced solely by the separation between morphologies. Together with the opposing relationships of the mean angle with *C*_33_^beq^ and *C*_66_^beq^, they show that changes in local geometry accompany a redistribution of stiffness between the axial and in-plane directions.

### 3.7. Coordination-Dependent Bonding, Electronic Structure, and Elastic Relationships

**Figure 7:**
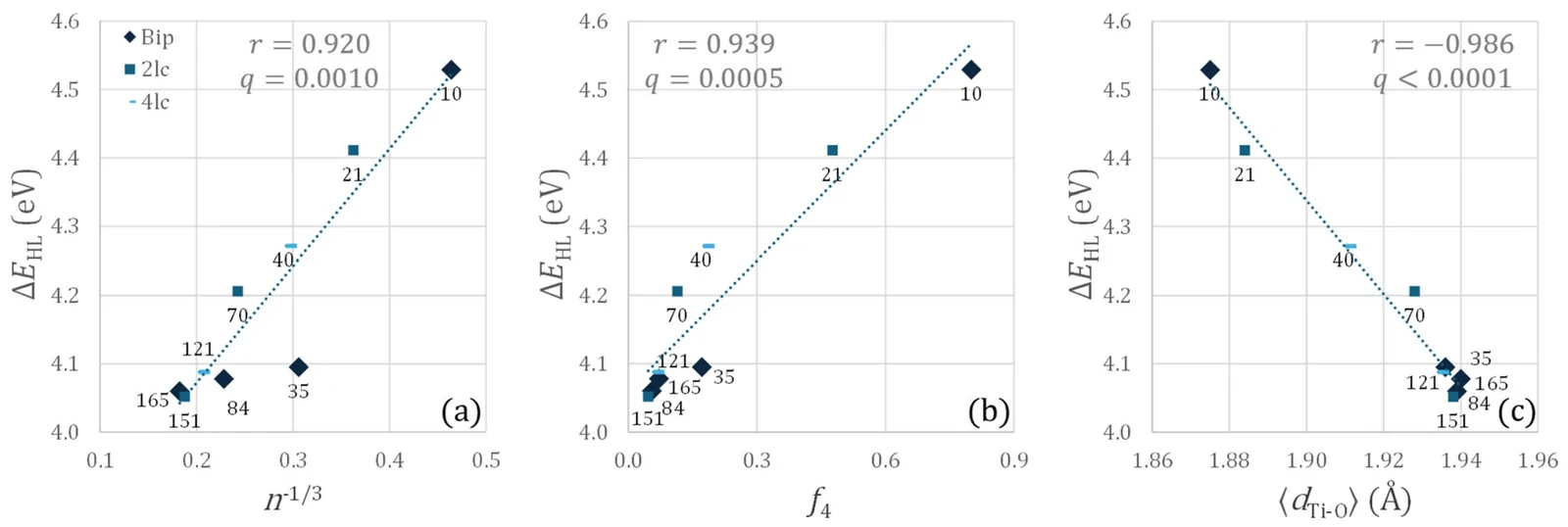
Structural correlations with the HOMO–LUMO gap, Δ*E*_HL_, of anatase-derived TiO_2_ nanoclusters: Δ*E*_HL_ versus (**a**) inverse cluster size, *n*^−1/3^, (**b**) the fraction of 4-coordinate Ti atoms, *f*_4_, and (**c**) the mean Ti–O bond length, ⟨*d*_Ti-O_⟩. Diamonds, squares, and rectangles identify the bipyramidal, 2-layer-cut, and 4-layer-cut clusters, respectively. Dotted lines show linear least-squares fits across the nine clusters, which are labeled by the number of Ti atoms, *n*. The reported *r* values are Pearson correlation coefficients; *q* values are the corresponding two-sided *p* values adjusted by the Benjamini–Hochberg procedure within the predefined family of structural descriptors tested against the HOMO–LUMO gap.

**Figure 8:**
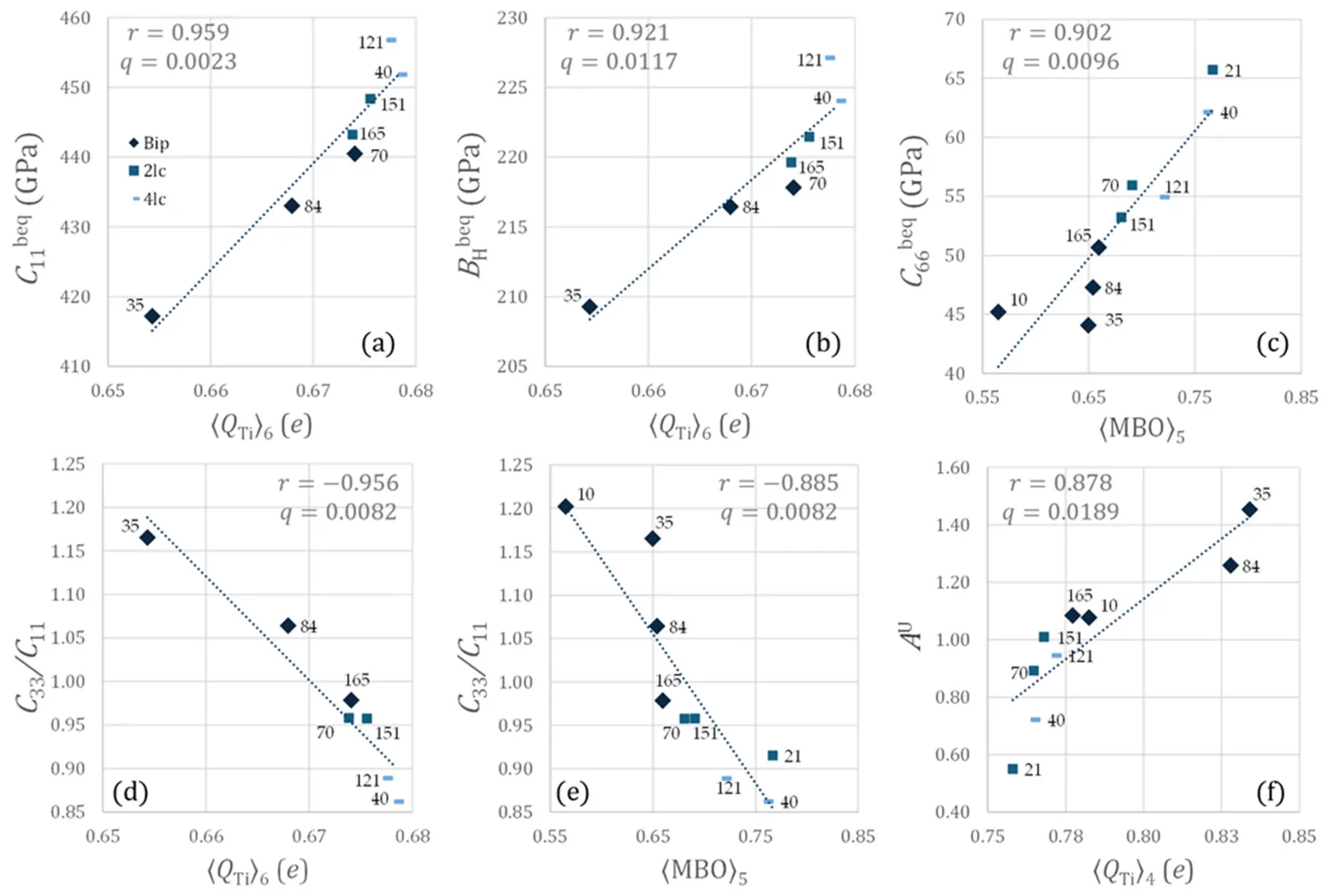
Correlations between coordination-resolved electronic descriptors and the effective elastic response of anatase-derived TiO_2_ nanoclusters: (**a**) *C*_11_^beq^ and (**b**) *B*_H_^beq^ versus the mean Hirshfeld charge of 6-coordinate Ti, ⟨*Q*_Ti_⟩_6_; (**c**) *C*_66_^beq^ versus the mean Mayer Ti–O bond order of 5-coordinate Ti, ⟨MBO⟩_5_; *C*_33_/*C*_11_ versus (**d**) ⟨*Q*_Ti_⟩_6_ and (**e**) ⟨MBO⟩_5_; and (**f**) the universal elastic anisotropy index, *A*^U^, versus the mean Hirshfeld charge of 4-coordinate Ti, ⟨*Q*_Ti_⟩_4_. Diamonds, squares, and rectangles identify the bipyramidal, 2-layer-cut, and 4-layer-cut clusters, respectively. Dotted lines show linear least-squares fits, and clusters are labeled by the number of Ti atoms, *n*. Panels (**a**,**b**,**d**) contain the seven clusters with 6-coordinate Ti; the remaining panels contain all nine clusters. The reported *r* values are Pearson correlation coefficients; *q* values are the corresponding two-sided *p* values adjusted by the Benjamini–Hochberg procedure within the predefined family of electronic descriptors tested against each elastic target.

**Figure 9:**
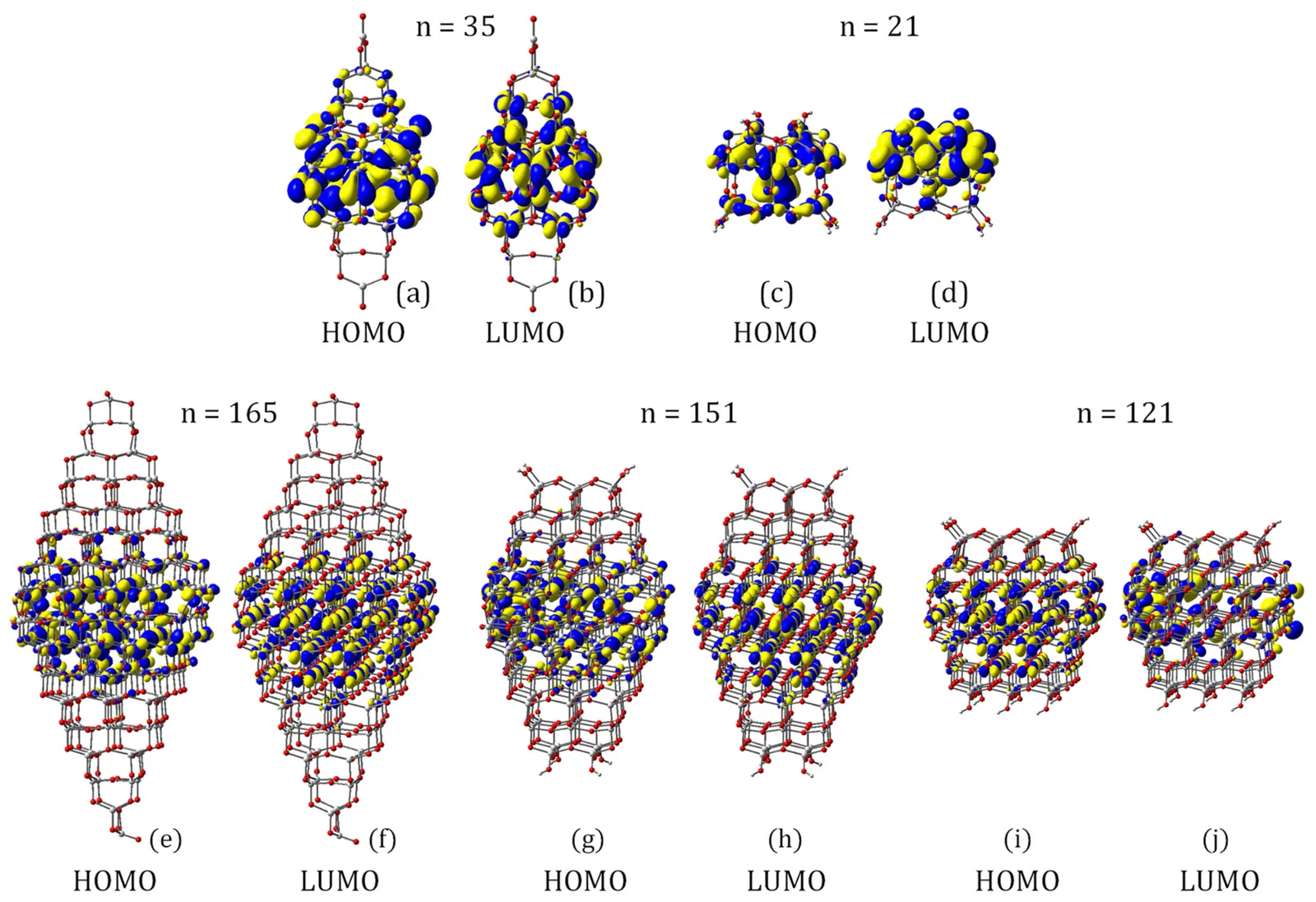
Frontier molecular orbital isosurfaces of selected anatase-derived TiO_2_ nanoclusters. (**Top row**) HOMO and LUMO of the *n* = 35 bipyramidal, (**a**,**b**), and *n* = 21 2-layer-cut, (**c**,**d**), clusters, illustrating the effect of layer cutting at comparable cluster size. (**Bottom row**) HOMO and LUMO of the three largest clusters, *n* = 165 bipyramidal, (**e**,**f**), *n* = 151 2-layer-cut, (**g**,**h**), and *n* = 121 4-layer-cut, (**i**,**j**), illustrating the evolution of orbital character across morphologies at large *n*. Ti, O, and H atoms are shown in dark gray, red, and light gray, respectively.

The electronic analysis considers coordination-resolved Mayer Ti–O bond orders and Hirshfeld Ti charges, the HOMO–LUMO gap, and the spatial distribution of the frontier molecular orbitals. Table 6 summarizes the coordination-resolved electronic descriptors, with the corresponding means and standard deviations reported in Table S6. Figure 7 examines the relationships of cluster size, 4-coordinate Ti fraction, and mean Ti–O bond length with the HOMO–LUMO gap. Figure 8 then relates coordination-resolved charges and bond orders to bulk-equivalent elastic properties and normalization-independent anisotropy measures, while Figure 9 shows the HOMO and LUMO isosurfaces for representative parent and cut clusters.

The coordination-resolved descriptors show systematic differences among the Ti environments. The mean Mayer Ti–O bond order is highest for bonds involving 4-coordinate Ti (0.80–0.93), whereas the 5- and 6-coordinate classes occupy lower, partly overlapping ranges (0.56–0.77 and 0.58–0.62, respectively). The mean positive Hirshfeld charge shows a clearer coordination ordering, increasing from 0.65 to 0.68 *e* for 6-coordinate Ti to 0.71–0.74 *e* for 5-coordinate Ti and 0.76–0.83 *e* for 4-coordinate Ti. Thus, 4-coordinate Ti sites are characterized by both the largest mean Ti–O bond orders and the highest positive Ti charges, whereas the distinction between the 5- and 6-coordinate environments is more pronounced in the charges than in the bond orders.

The calculated HOMO–LUMO gaps range from 4.052 to 4.529 eV; the individual values, together with the corresponding structural and coordination descriptors, are reported in Table S4. The HOMO–LUMO gap increases with inverse cluster size (Figure 7a) and with the fraction of 4-coordinate Ti atoms. An even stronger inverse relationship is found with the mean Ti–O bond length: clusters with shorter mean Ti–O bonds have larger gaps. Together, these results show that the gap is associated not only with cluster size but also with the accompanying evolution of surface coordination and bond contraction. Among the examined structural descriptors, the mean Ti–O bond length has the strongest correlation with the HOMO–LUMO gap.

Coordination-resolved electronic descriptors also reveal strong associations with the bulk-equivalent elastic response. Among the seven clusters containing 6-coordinate Ti, the mean Hirshfeld charge ⟨*Q*_Ti_⟩_6_ is positively correlated with both *C*_11_^beq^ and *B*_H_^beq^ (Figure 8). In addition, the mean Ti–O bond order associated with 5-coordinate Ti, ⟨MBO⟩_5_ increases with *C*_66_^beq^. Thus, greater positive charge on 6-coordinate Ti and stronger bonding around 5-coordinate Ti are associated with a stiffer bulk-equivalent response along distinct deformation modes.

The same electronic descriptors are also associated with the redistribution of stiffness among directions. For the seven clusters containing 6-coordinate Ti, ⟨*Q*_Ti_⟩_6_ is strongly negatively correlated with *C*_33_/*C*_11_. The ratio likewise decreases as ⟨MBO⟩_5_ increases, whereas the universal elastic anisotropy index increases with ⟨*Q*_Ti_⟩_4_. These relationships indicate that coordination-dependent charge redistribution and Ti–O bonding are associated not only with the magnitude of the bulk-equivalent response but also with its directional anisotropy.

A complementary inverse association is observed between ⟨MBO⟩_4_ and *A*^U^ (*r* = −0.818, *q* = 0.0193; Table S5). However, its direction is not reproduced within both morphological subsets, indicating that this whole-set relationship should be interpreted cautiously.

Figure 9 shows both the atomic-orbital character and the spatial localization of the frontier states. The HOMO has predominantly O 2*p* character, as indicated by the *p*-like lobes centered on O atoms, whereas the LUMO has predominantly Ti 3*d* character, with *d*-like lobes centered on Ti atoms. The O 2*p*–Ti 3*d* frontier-state character is well established [24] and is retained across the five representative clusters, even though their spatial distributions change with cluster geometry. In the 35→21 comparison, both orbitals evolve from distributions extending along the bipyramidal framework to more compact distributions concentrated around the cut structure, with the LUMO showing particularly strong localization near the exposed surface. In the *n* = 165 family, removal of two and four layers progressively shifts the frontier-orbital isosurfaces toward the cut faces. Thus, apical cutting primarily redistributes the HOMO and LUMO spatially while preserving their characteristic orbital nature.

Taken together, these results identify interconnected associations among structure, electronic descriptors, and effective elasticity. Decreasing cluster size is accompanied by changes in Ti coordination and Ti–O bond lengths, with larger *f*_4_ and shorter mean Ti–O bonds associated with wider HOMO–LUMO gaps. Coordination-resolved charges and bond orders are, in turn, strongly associated with both the magnitude and directional distribution of the bulk-equivalent elastic response. The electronic structure therefore provides a link between the evolving local Ti–O environment and the effective stiffness and anisotropy of the nanoclusters. These relationships provide the basis for the mechanistic interpretation developed in Section 4.

## 4. Discussion

The present results support three connected structure–property narratives for finite TiO_2_ clusters. First, the effective elastic response depends on both the reference-volume convention and the evolving cluster structure. Under bulk-equivalent normalization, decreasing size does not produce universal stiffening; instead, morphology, aspect ratio, and local Ti–O geometry are associated with the redistribution of stiffness among deformation modes. Second, structural changes are linked to the electronic response: increasing *f*_4_ and decreasing mean Ti–O bond length accompany widening of the HOMO–LUMO gap, with the mean bond length providing the strongest correlation. Third, coordination-resolved Ti charges and Ti–O bond orders are associated with both bulk-equivalent stiffness and normalization-independent anisotropy. Together, these findings connect cluster construction, local atomic structure, electronic organization, and effective elasticity, while remaining exploratory because size, morphology, and coordination are partly coupled within the nine-cluster dataset.

### 4.1. Reference Volume and the Meaning of Effective Cluster Elasticity

The primary mechanical information obtained for a finite cluster is the curvature of its energy with respect to strain. This curvature is uniquely determined by the energy–strain calculation, whereas its conversion into an effective elastic coefficient in pressure units requires a reference volume. The resulting coefficient therefore combines two elements: the intrinsic energetic response of the cluster to deformation and the volume convention used to express that response per unit volume.

This distinction directly affects the interpretation of size-dependent behavior. Changing the reference volume rescales all dimensional effective elastic coefficients and moduli of a given cluster by the same positive factor, but that factor varies among clusters because the ratios between the volume definitions depend on size and morphology. Consequently, the trends can reverse sign: the correlation of *B*_H_ with *n*^−1/3^ changes from *r* = 0.980 under *V*_core_ to *r* = −0.782 under *V*_beq_, while its correlation with *f*_4_ changes from *r* = 0.949 to *r* = −0.778. Because the underlying energy–strain curvatures are identical, these reversals arise from the cluster-dependent volume scaling, and the apparent trends cannot be attributed uniquely to an intrinsic change in stiffness. Under *V*_beq_, both Pearson correlations remain below the multiple-comparison threshold after Benjamini–Hochberg adjustment (*q* = 0.0377), but their rank-based support is weaker: *ρ* = −0.633, Spearman *p* = 0.0671 for *n*^−1/3^, and *ρ* = −0.567, Spearman *p* = 0.112 for *f*_4_. Thus, bulk-equivalent normalization does not support universal stiffening with decreasing size, while the apparent negative trends should also be interpreted cautiously.

The bulk-equivalent convention provides a common compositional reference by assigning the anatase volume per TiO_2_ formula unit to each cluster formula unit. It is consequently the most transparent convention for comparisons across the present series. The convex-hull volumes remain useful geometric descriptors of the particle core or of the complete hydroxylated structure, but the corresponding effective coefficients must be interpreted together with those geometric definitions. By contrast, dimensionless quantities such as *C*_33_/*C*_11_, *B*_H_/*G*_H_, ν, and *A*^U^ are unchanged by uniform volume rescaling and provide normalization-invariant descriptions of the relative elastic response.

### 4.2. Size Dependence and Directional Elastic Response to Apical Cutting

Under bulk-equivalent normalization, decreasing cluster size does not produce a single stiffness trend. The axial, transverse, coupling, and shear coefficients evolve differently, and several exhibit nonmonotonic behavior within the bipyramidal family. This component-specific response indicates that size reduction changes the directional organization of the Ti–O framework rather than simply making the entire cluster uniformly stiffer or softer. The comparison with crystalline anatase leads to the same conclusion: some effective coefficients exceed their crystalline counterparts, whereas others remain lower throughout the cluster series.

The parent–cut comparison gives the clearest view of the effect, since the two structures of a pair differ by the deliberate removal of a fixed number of atomic layers, rather than by overall size. In every pair, cutting lowers the axial stiffness relative to the in-plane one and raises in-plane coupling and shear relative to it. These are ratios between coefficients of the same cluster, so no choice of reference volume can create the effect or remove it. Cutting therefore does not make a particle globally stiffer or softer: it moves stiffness out of the elongated direction. The axially dominated response of a tall bipyramid gives way to a more balanced one in the flatter cut cluster, where in-plane coupling and shear weigh more heavily in the tensor. This follows naturally from the removal of the apical caps, the loss of elongation, and the relaxation and hydroxyl passivation of the newly exposed surfaces.

Spatial mapping of the altered Ti environments suggests a structural interpretation of this redistribution. In the parent bipyramids, the most asymmetric 4-coordinate Ti sites occupy the axial apices and contain one unusually long Ti–O contact directed inward along the long axis. Apical cutting removes these sites together with the surrounding subapical framework and exposes new terminal layers. Hydroxylation satisfies part of the resulting local coordination demand, but terminal Ti–OH bonds do not recreate the removed framework-spanning Ti–O–Ti connections. Consistently, cutting shortens the mean Ti–O distance, narrows its distribution, and increases the mean bond order around 4-coordinate Ti, while nevertheless lowering *C*_33_/*C*_11_. The remaining local bonds can therefore become shorter and electronically stronger while axial stiffness decreases relative to in-plane stiffness. This coexistence suggests that directional framework connectivity contributes to the response alongside local bond strength. The present comparisons support this interpretation but do not independently isolate a connectivity contribution.

The magnitude of this redistribution generally becomes larger for deeper cutting and for smaller parent clusters, where removing a given number of atomic layers represents a greater modification of the original structure. The near-doubling of *C*_12_/*C*_11_ for 35→21 (94.7% increase) should be read together with its starting point: *C*_12_ is the smallest coefficient of the parent, so a modest absolute change looks large in relative terms and does not by itself make transverse coupling a dominant term in the tensor. Across the five pairs, though, the direction of the shift is the same, so within this family apical cutting changes the balance between axial, transverse and shear response in a predictable direction.

### 4.3. Shape and Local Distortion as Structural Correlates of Effective Elasticity

The bulk-equivalent results show that local geometry is associated with both the overall resistance to compression and individual deformation modes. *B*_H_^beq^ decreases as the Ti–O bond-length dispersion increases, linking greater local bond-length heterogeneity with a softer bulk-equivalent response. By contrast, the mean O–Ti–O angle has opposite relationships with two individual coefficients: *C*_33_^beq^ increases, whereas *C*_66_^beq^ decreases. Local structural evolution therefore does not shift every elastic component uniformly but accompanies a redistribution between axial and shear resistance.

The normalization-invariant quantities clarify how overall cluster shape relates to the distribution of the elastic response. The universal anisotropy index, *A*^U^, is strongly positively correlated with the aspect ratio, *AR*, and the relationship retains the same direction within both the bipyramidal and cut-cluster subsets. The axial-to-in-plane stiffness ratio *C*_33_/*C*_11_ is also positively correlated with the aspect ratio *AR* when considering the complete dataset. However, this relationship is negative within the bipyramidal subset and positive within the cut-cluster subset. Thus, elongation is consistently associated with greater overall anisotropy, whereas its association with the axial-to-in-plane stiffness ratio is conditioned by morphology.

Local geometric descriptors refine this interpretation, providing a more consistent account of the axial-to-in-plane partitioning of stiffness. The axial-to-in-plane stiffness ratio *C*_33_/*C*_11_ increases with both the mean O–Ti–O angle and the Ti–O bond-length dispersion, with both relationships retaining the same direction within the bipyramidal and cut-cluster subsets. The mean angle characterizes the average geometry of the Ti coordination framework, whereas the bond-length dispersion reflects its local heterogeneity. Their robust associations with *C*_33_/*C*_11_ indicate that the directional elastic response is closely connected to the local organization of the Ti–O network.

The structural correlations therefore distinguish several related aspects of the elastic response. Bond-length heterogeneity is associated with the bulk-equivalent resistance to compression. Changes in mean coordination geometry accompany opposing shifts in axial and shear coefficients; global shape is associated most consistently with overall anisotropy; and local bond-length and bond-angle descriptors track the axial-to-in-plane partitioning of stiffness. Apical cutting modifies all these structural levels simultaneously by changing the external proportions of the cluster and reorganizing its Ti–O network. The resulting elastic response reflects their combined evolution rather than a single size-dependent stiffening or softening mechanism.

### 4.4. Coordination-Dependent Bonding, Electronic Structure, and Elastic Response

The coordination-resolved electronic descriptors provide an atomistic view of how the Ti–O network changes across the cluster series. As the Ti coordination number decreases from six to four, the mean Mayer Ti–O bond order increases and the Ti atoms acquire a larger positive Hirshfeld charge. Lower-coordinate Ti environments are therefore characterized by stronger individual Ti–O bond interactions and greater charge separation. This electronic reorganization accompanies the structural replacement of bulk-like 6-coordinate sites by surface 4-coordinate sites as cluster size decreases or the bipyramidal structure is cut.

The HOMO–LUMO gap increases with both inverse cluster size and the fraction of 4-coordinate Ti, but its strongest structural relationship is the inverse correlation with the mean Ti–O bond length. The coordination composition therefore describes the gap evolution more directly than atom count alone, while the still stronger bond-length relationship identifies contraction of the Ti–O network as the structural feature most closely associated with gap widening. Size reduction, increased abundance of low-coordinate surface sites, and shorter mean Ti–O bonds evolve together in the present cluster set, so their individual contributions cannot yet be separated mechanistically. Nevertheless, the combined results show that the gap reflects the evolving local Ti–O environment rather than cluster size alone.

The present HOMO–LUMO gaps are of comparable magnitude to the 4.38–5.15 eV obtained previously for hydroxyl-terminated anatase-derived clusters using B3LYP/LANL2DZ in water [40]. That study also found an overall decrease in the gap with increasing cluster size. Differences in cluster morphology, termination, basis set and environment preclude a direct numerical correspondence. These frontier-orbital gaps should also be distinguished from optical transition energies, as discussed in Ref. [40].

The frontier orbitals support this coordination- and shape-dependent picture. Across the investigated clusters, the HOMO retains predominantly O 2*p* character and the LUMO predominantly Ti 3*d* character, while their spatial distributions change with size and cutting. Apical removal redistributes the frontier-orbital density toward the remaining surfaces and cut regions without changing the principal atomic-orbital character of the states.

Coordination-resolved electronic descriptors are also strongly associated with the magnitude of the bulk-equivalent elastic response. Among the seven clusters containing 6-coordinate Ti, a larger mean positive Ti charge is associated with higher *C*_11_^beq^ and *B*_H_^beq^. Stronger mean Ti–O bonding around 5-coordinate Ti is likewise associated with higher *C*_66_^beq^. These relationships connect the electronic environments of the more highly coordinated Ti sites with distinct components of the effective stiffness.

The same descriptors also track the directional distribution of stiffness. Both ⟨*Q*_Ti_⟩_6_ and ⟨MBO⟩_5_ are negatively associated with *C*_33_/*C*_11_, whereas ⟨*Q*_Ti_⟩_4_ is positively associated with the universal anisotropy index *A*^U^. An inverse whole-set relationship between ⟨MBO⟩_4_ and *A*^U^ is less robust because its direction is not retained within both morphology subsets. Coordination-dependent charge redistribution and Ti–O bonding are therefore associated with both effective stiffness and anisotropy, although a mechanistic interpretation will require a larger set in which size, morphology, and coordination vary more independently.

### 4.5. Implications, Limitations, and Outlook

The procedure used here is not specific to anatase and can be applied to other finite nanostructures, although it has so far been tested on one oxide and one family of morphologies. The energy–strain curvature should be retained as the primary volume-independent result, while every dimensional effective coefficient or modulus should be reported together with its reference-volume definition. Three practices together make it possible to distinguish energetic changes from normalization effects: a composition-based volume for comparisons, geometric volumes for complementary interpretation, and dimensionless invariants for normalization-independent analysis. This distinction is essential whenever the surface contribution to the assigned volume varies with particle size, shape, or passivation.

Three connected classes of relationships emerge from the calculations. Cluster shape and local Ti–O distortion are associated with the magnitude and directional distribution of the elastic response; coordination composition and mean Ti–O bond length are associated with the HOMO–LUMO gap; and coordination-resolved charges and bond orders are associated with bulk-equivalent stiffness and elastic anisotropy. These relationships suggest a pathway from structural modification, through electronic reorganization, to mechanical response. They do not yet establish a causal sequence, because size, shape, coordination, and passivation are partly coupled in the present structures. Separating them as design variables will require cluster sets in which each factor is varied independently.

The present dataset contains nine clusters in which size, morphology, composition, and hydroxyl passivation are partly coupled; three extensions would sharpen the analysis. First, a broader set of structures with matched size and composition would allow these contributions to be separated more completely. Second, calculation of the full elastic tensor across the cluster series would extend the present targeted symmetry tests to the untested structures and determine the coupling terms retained at zero in the expanded reconstruction. Third, because the cluster coefficients are clamped-ion while the ionic-relaxation treatment varies among the published bulk studies and is not always reported explicitly, future calculations that allow internal relaxation while maintaining each prescribed cluster deformation, together with bulk calculations at the same electronic-structure level, would quantify this contribution and provide a more controlled cluster–bulk comparison. Attempts to establish a periodic bulk reference in Gaussian encountered numerical termination or SCF nonconvergence with the tested larger basis sets. No converged bulk elastic reference consistent with the cluster treatment was obtained.

Comparison with experimental measurements likewise requires attention to the deformation conditions and the definition of the reported modulus. High-pressure X-ray diffraction measurements yielded bulk moduli of 204(8) GPa for rice-shaped anatase nanoparticles (3.8 × 5.0 nm) and 319(20) GPa for rod-shaped nanoparticles (3.5 × 21.0 nm) [17]. These measurements demonstrate the relevance of particle geometry to compressibility and provide experimental context for the directional response examined here. However, the different particle sizes and the distinction between experimental compression and prescribed clamped-ion deformation limit the comparison to qualitative observations.

Extension to larger clusters, alternative facets and morphologies, and other oxide nanostructures will establish how broadly the identified relationships apply. With larger systematically designed datasets, multivariable and predictive models could quantify the separate contributions of size, shape, coordination, passivation, and local distortion. The methodology developed here provides that foundation, summarized in Section 5.

## 5. Conclusions

Effective elastic coefficients, moduli, and anisotropy measures were determined from clamped-ion energy–strain DFT calculations for nine anatase-derived TiO_2_ nanoclusters, using a tetragonal-form model. The results distinguish changes in the deformation response from apparent stiffness trends introduced by volume normalization.

The analysis demonstrates that the energy curvature is the fundamental volume-independent result, whereas every dimensional effective elastic quantity depends on the reference volume used to express it in pressure units.

The same TiO_2_ energy–strain data can produce opposite apparent size trends when different reference volumes are used. Convex-hull normalization produces apparent stiffening toward smaller clusters, whereas bulk-equivalent normalization does not reveal universal size-dependent stiffening. Thus, an apparent increase in nanocluster stiffness can reflect the volume assigned to the structure as well as its deformation response. Reporting the volume convention is essential when comparing absolute elastic values; stiffness ratios and dimensionless anisotropy measures provide complementary comparisons independent of that convention.

Across all five matched parent–cut pairs, cutting and passivating the apices reduces axial stiffness relative to in-plane stiffness and increases relative in-plane coupling and shear. This directional redistribution persists under every volume convention. The universal anisotropy index also increases with aspect ratio, including within the bipyramidal and cut families, linking the balance of the elastic response to cluster geometry.

Local structural and electronic descriptors track different aspects of this response. Under bulk-equivalent normalization, the Hill bulk modulus decreases with increasing Ti–O bond-length dispersion. The HOMO–LUMO gap widens as mean Ti–O bonds shorten and the fraction of 4-coordinate Ti increases, while coordination-resolved charges and bond orders correlate with stiffness and anisotropy. These associations remain exploratory because size, morphology, coordination, and passivation vary together in the present dataset.

The parent–cut comparisons reveal the central physical finding: Ti–O bonds become shorter, more regular, and electronically stronger around 4-coordinate Ti, while axial stiffness decreases relative to in-plane stiffness. Local bond strengthening therefore does not translate into uniform stiffening of the complete structure. Stronger local bonds do not necessarily stiffen every direction of the nanocluster.

These results establish a transparent framework for extending effective-elasticity calculations to other finite nanostructures, resting on five practices: reporting the energy curvatures, defining the reference volume explicitly, comparing alternative volume conventions, using normalization-invariant quantities, and constructing matched structural comparisons.

## Figures and Tables

**Table 1:** Structural descriptors of the optimized anatase-derived TiO_2_ nanoclusters: number of Ti atoms, *n*, fractions *f*_k_ of Ti atoms with coordination number *k*, the aspect ratio, *AR*, and the relative difference between the transverse cluster dimensions *δ_xy_*. The remaining structural and electronic descriptors are reported in the Supplementary Material (Table S4).

Cluster	*n*	Morphology	*f*_4_	*f*_5_	*f*_6_	*AR*	*δ_xy_* (%)
Ti_165_O_330_	165	bipyramidal	0.055	0.515	0.430	2.457	0.66
Ti_151_O_298_(OH)_8_	151	2-layer cut	0.046	0.510	0.444	1.582	0.74
Ti_121_O_236_(OH)_12_	121	4-layer cut	0.058	0.537	0.405	0.993	1.31
Ti_84_O_168_	84	bipyramidal	0.071	0.619	0.310	2.694	0.00
Ti_70_O_136_(OH)_8_	70	2-layer cut	0.114	0.571	0.314	1.287	2.56
Ti_40_O_74_(OH)_12_	40	4-layer cut	0.175	0.725	0.100	0.548	8.60
Ti_35_O_70_	35	bipyramidal	0.171	0.686	0.143	2.757	0.23
Ti_21_O_38_(OH)_8_	21	2-layer cut	0.476	0.524	0.000	0.776	9.64
Ti_10_O_20_	10	bipyramidal	0.800	0.200	0.000	2.060	6.63

**Table 2:** Internal consistency checks for the extraction of the bulk-equivalent effective elastic coefficients for anatase-derived TiO_2_ nanoclusters. The Voigt bulk modulus $B_{V,\mathrm{calc}}^{\mathrm{beq}}$, reconstructed from the six reported coefficients, is compared with $B_{{V,D}_{8}}^{\mathrm{beq}}$, obtained directly from the isotropic deformation *D*_8_. For the bipyramidal clusters, $C_{11,\mathrm{calc}}^{\mathrm{beq}}$, obtained from the combined *D*_1_ and *D*_2_ deformations, is compared with $C_{11,D_{7}}^{\mathrm{beq}}$, obtained independently from the uniaxial deformation *D*_7_. Relative differences are reported as signed percentages, calculated from the unrounded values as 100 × (*C*_direct_ − *C*_calc_)/*C*_calc_.

*n*	Morphology	$B_{V,\mathrm{calc}}^{\mathrm{beq}}$	$B_{{V,D}_{8}}^{\mathrm{beq}}$	Difference (%)	$C_{11,\mathrm{calc}}^{\mathrm{beq}}$	$C_{11,D_{7}}^{\mathrm{beq}}$	Difference (%)
165	bipyramidal	218.8	219.0	0.06	440.5	437.6	−0.65
151	2-layer cut	222.3	222.9	0.27	—	—	—
121	4-layer cut	227.7	224.1	−1.57	—	—	—
84	bipyramidal	218.3	216.9	−0.67	433.0	438.5	1.27
70	2-layer cut	220.3	222.3	0.90	—	—	—
40	4-layer cut	224.2	225.1	0.39	—	—	—
35	bipyramidal	212.2	212.3	0.02	417.2	419.7	0.61
21	2-layer cut	214.9	223.5	3.98	—	—	—
10	bipyramidal	204.4	204.1	−0.13	371.7	386.6	4.03

**Table 3:** Bulk-equivalent effective elastic coefficients, $C_{\mathrm{ij}}^{\mathrm{beq}}$, in GPa, of anatase-derived TiO_2_ nanoclusters, obtained using the reference volume *V*_beq_. The bulk row gives the range spanned by sixteen published DFT elastic tensors for crystalline anatase, Refs. [23,26,27,28,29,30,31,32,33,34,35].

*n*	Morphology	$C_{11}^{\mathrm{beq}}$	$C_{12}^{\mathrm{beq}}$	$C_{13}^{\mathrm{beq}}$	$C_{33}^{\mathrm{beq}}$	$C_{44}^{\mathrm{beq}}$	$C_{66}^{\mathrm{beq}}$
165	bipyramidal	440.5	47.0	140.9	431.0	100.2	50.7
151	2-layer cut	448.4	49.0	144.1	429.4	102.7	53.2
121	4-layer cut	456.8	49.7	157.5	406.1	102.9	55.0
84	bipyramidal	433.0	45.6	136.7	460.9	97.5	47.3
70	2-layer cut	443.2	53.3	141.3	424.6	104.2	56.0
40	4-layer cut	451.8	57.8	152.3	389.6	101.6	62.2
35	bipyramidal	417.2	40.3	127.2	486.2	94.1	44.1
21	2-layer cut	435.3	81.8	125.3	398.5	114.4	65.7
10	bipyramidal	371.7	50.7	137.0	446.8	106.2	45.2
Bulk	minimum	309	112	134	172	16	47
	maximum	399	179	184	251	79	83

**Table 4:** Bulk-equivalent Hill moduli and normalization-invariant elastic measures of the anatase-derived TiO_2_ nanoclusters. The bulk modulus $B_{H}^{\mathrm{beq}},$ shear modulus $G_{H}^{\mathrm{beq}}$, and Young’s modulus *E*^beq^ are given in GPa. Poisson’s ratio *ν*, the Pugh ratio *B*_H_/*G*_H_ and the universal elastic anisotropy index *A*^U^ are dimensionless. The final three columns give the normalization-invariant ratios *C*_33_/*C*_11_, *C*_12_/*C*_11_, and *C*_66_/*C*_11_. The bulk row gives the range of the same quantities computed here from each of those sixteen tensors with the same Voigt–Reuss–Hill procedure (Refs. [23,26,27,28,29,30,31,32,33,34,35]); they are not the moduli reported in the original papers.

*n*	Morphology	$B_{H}^{\mathrm{beq}}$	$G_{H}^{\mathrm{beq}}$	*E* ^beq^	*ν*	*B*_H_/*G*_H_	*A* ^U^	*C*_33_/*C*_11_	*C*_12_/*C*_11_	*C*_66_/*C*_11_
165	bipyramidal	217.8	105.5	272.5	0.292	2.064	1.085	0.978	0.107	0.115
151	2-layer cut	221.5	107.8	278.3	0.291	2.054	1.010	0.958	0.109	0.119
121	4-layer cut	227.1	106.6	276.6	0.297	2.130	0.946	0.889	0.109	0.120
84	bipyramidal	216.5	104.1	269.3	0.293	2.078	1.259	1.064	0.105	0.109
70	2-layer cut	219.6	109.0	280.6	0.287	2.015	0.892	0.958	0.120	0.126
40	4-layer cut	224.0	107.9	278.9	0.293	2.077	0.722	0.862	0.128	0.138
35	bipyramidal	209.3	102.1	263.5	0.290	2.050	1.453	1.165	0.097	0.106
21	2-layer cut	214.9	115.3	293.5	0.272	1.863	0.550	0.915	0.188	0.151
10	bipyramidal	200.6	99.8	256.8	0.287	2.010	1.077	1.202	0.137	0.122
Bulk	minimum	174.4	35.6	99.9	0.324	2.50	0.30	—	—	—
	maximum	218.6	75.6	203.4	0.408	5.11	3.08	—	—	—

**Table 5:** Dependence of the Hill bulk modulus *B*_H_ and its correlations with cluster size and Ti coordination for the three volume reference conventions used and all anatase-derived TiO_2_ nanoclusters. The table gives the range of *B*_H_ across the nine clusters and Pearson correlation coefficients *r* with inverse cluster size *n*^−1/3^ and the fraction of 4-coordinate Ti atoms *f*_4_. The *p* values are two-sided unadjusted values for Pearson *r*, whereas *ρ* is the Spearman rank coefficient used as a robustness measure. The corresponding Benjamini–Hochberg-adjusted *q* values, Spearman *p* values, leave-one-out ranges, and within-family coefficients are given in the Supplementary Material (Table S5).

Volume Reference	*B*_H_ Range (GPa)	*r* (*B*_H_, *n*^−1/3^)	*p*	*ρ* (*B*_H_, *n*^−1/3^)	*r* (*B*_H_, *f*_4_)	*p*	*ρ* (*B*_H_, *f*_4_)
*V*_beq_	200.6–227.1	−0.782	0.0128	−0.633	−0.778	0.0136	−0.567
*V*_core_	281.9–554.7	0.980	<0.0001	0.967	0.949	<0.0001	0.967
*V*_tot_	255.7–554.7	0.743	0.0219	0.250	0.765	0.0162	0.167

**Table 6:** Coordination-resolved mean Mayer Ti–O bond orders ⟨MBO⟩*_k_* and mean Hirshfeld charges ⟨*Q*⟩*_k_* of the Ti atoms in coordination class *k*, at the same level of theory as the structural calculations. Bond orders are grouped by the coordination number of the Ti atom of the bond. A dash marks a coordination class that is absent from the cluster. The complete cluster-resolved values and standard deviations are given in Table S6.

*n*	⟨MBO⟩_6_	⟨MBO⟩_5_	⟨MBO⟩_4_	⟨*Q*⟩_6_ (*e*)	⟨*Q*⟩_5_ (*e*)	⟨*Q*⟩_4_ (*e*)
165	0.5937	0.6594	0.8046	0.6741	0.7368	0.7772
151	0.5890	0.6808	0.8237	0.6756	0.7321	0.768
121	0.5982	0.7177	0.8246	0.6772	0.7288	0.7708
84	0.6170	0.6539	0.8203	0.6679	0.7373	0.8279
70	0.6125	0.6908	0.8418	0.6738	0.7276	0.7647
40	0.5815	0.7585	0.9154	0.6783	0.721	0.764
35	0.6201	0.6496	0.8182	0.6543	0.7362	0.8341
21	—	0.7667	0.9292	—	0.7137	0.758
10	—	0.5649	0.8189	—	0.7177	0.7825

## Data Availability

The data presented in this study, including the extracted elastic coefficients, structural descriptors, and correlation analyses, are available within the article and its Supplementary Materials. The optimized cluster geometries generated in this study are available from the corresponding author upon reasonable request.

## Supplementary Materials

The following supporting information can be downloaded at: https://www.mdpi.com/article/10.3390/nano16211382/s1, Figure S1. Histogram of Ti–O pairwise distance distribution across the nine anatase TiO_2_ clusters; Figure S2. Ener-gy–strain curves for the eight homogeneous deformation modes; Table S1. Energy curvatures 2q_2_, their standard errors, R^2^ and the inner/outer curvature difference; Table S2. Cluster dimensions and the three reference volumes; Table S3. Effective coefficients and Hill moduli under the two convex-hull conventions; Figure S3. Effective elastic coefficients under the core convex-hull convention versus inverse cluster size; Figure S4. Total energy per TiO_2_ unit versus inverse cluster size for the stoichiometric bipyramidal clusters; Table S4. Local bonding descriptors, coordi-nation fractions, aspect ratio and HOMO–LUMO gap; Table S5. Statistics for every correlation quoted in the main text; Table S6. Coordination-resolved Mayer bond orders and Hirshfeld Ti charges; Table S7. Targeted tests of the tetragonal transverse equalities for the three 2-layer-cut clusters; Table S8. Reconstructed normal-strain block and measured shear diagonal entries, under core-hull normalization; Table S9. Aggregate quantities from the original six-coefficient form and expanded nine-coefficient sensitivity matrix; Table S10. Fit diagnostics for the added y-uniaxial and xz-shear modes; Table S11. Basis-and-geometry sensitivity for the n = 10 cluster; Table S12. Raw total energies and differences for the basis-and-geometry comparison; Section S1. Reconstruction of the expanded stiff-ness matrix and calculation of aggregate elastic quantities, Equations (S1)–(S7).

## Abbreviations

The following abbreviations are used in this manuscript:

DFT	Density functional theory
*AR*	Aspect ratio
*A*^U^	Universal anisotropy index
HOMO	Highest occupied molecular orbital
LUMO	Lowest unoccupied molecular orbital
MBO	Mayer bond order
