# Supplementary material for: Effective Elasticity of Anatase-Derived TiO2 Nanoclusters: Volume Normalization, Directional Response, and Structure–Property Correlations
Source: Nanomaterials (Basel). 2026 Oct 28;16(21):1382. doi: 10.3390/nano16211382 (PMC13765495; doi:10.3390/nano16211382)
Supplement: Supplementary file 1 [file nanomaterials-16-01382-s001.zip › nanomaterials-4613273-supplementary.pdf]

Supplementary Material

Effective Elasticity of Anatase-Derived TiO_2 Nanoclusters: Volume Normalization, Directional Response, and Structure–Property Correlations

Robert M. Solomon [1], Corneliu I. Oprea [2] and Mihai A. Gîrțu [2,*]

[1] Doctoral School of Physics, Faculty of Physics, University of Bucharest, 077125 Bucharest, Romania; robert-mihai.solomon@s.unibuc.ro
[2] Faculty of Applied Sciences and Engineering, Ovidius University of Constanța, 900527 Constanța, Romania; cornel.oprea@univ-ovidius.ro
* Correspondence: mihai.girtu@univ-ovidius.ro

Contents

• Figure S1. Histogram of Ti–O pairwise distance distribution across the nine anatase TiO_2 clusters.
• Figure S2. Energy–strain curves for the eight homogeneous deformation modes.
• Table S1. Energy curvatures $2q_2$, their standard errors, R^2 and the inner/outer curvature difference.
• Table S2. Cluster dimensions and the three reference volumes.
• Table S3. Effective coefficients and Hill moduli under the two convex-hull conventions.
• Figure S3. Effective elastic coefficients under the core convex-hull convention versus inverse cluster size.
• Figure S4. Total energy per TiO_2 unit versus inverse cluster size for the stoichiometric bipyramidal clusters.
• Table S4. Local bonding descriptors, coordination fractions, aspect ratio and HOMO–LUMO gap.
• Table S5. Statistics for every correlation quoted in the main text.
• Table S6. Coordination-resolved Mayer bond orders and Hirshfeld Ti charges.
• Table S7. Targeted tests of the tetragonal transverse equalities for the three 2-layer-cut clusters.
• Table S8. Reconstructed normal-strain block and measured shear diagonal entries, under core-hull normalization.
• Table S9. Aggregate quantities from the original six-coefficient form and expanded nine-coefficient sensitivity matrix.
• Table S10. Fit diagnostics for the added y-uniaxial and xz-shear modes.
• Table S11. Basis-and-geometry sensitivity for the $n = 10$ cluster.
• Table S12. Raw total energies and differences for the basis-and-geometry comparison.
• Section S1. Reconstruction of the expanded stiffness matrix and calculation of aggregate elastic quantities, Eqs. (S1)–(S7)

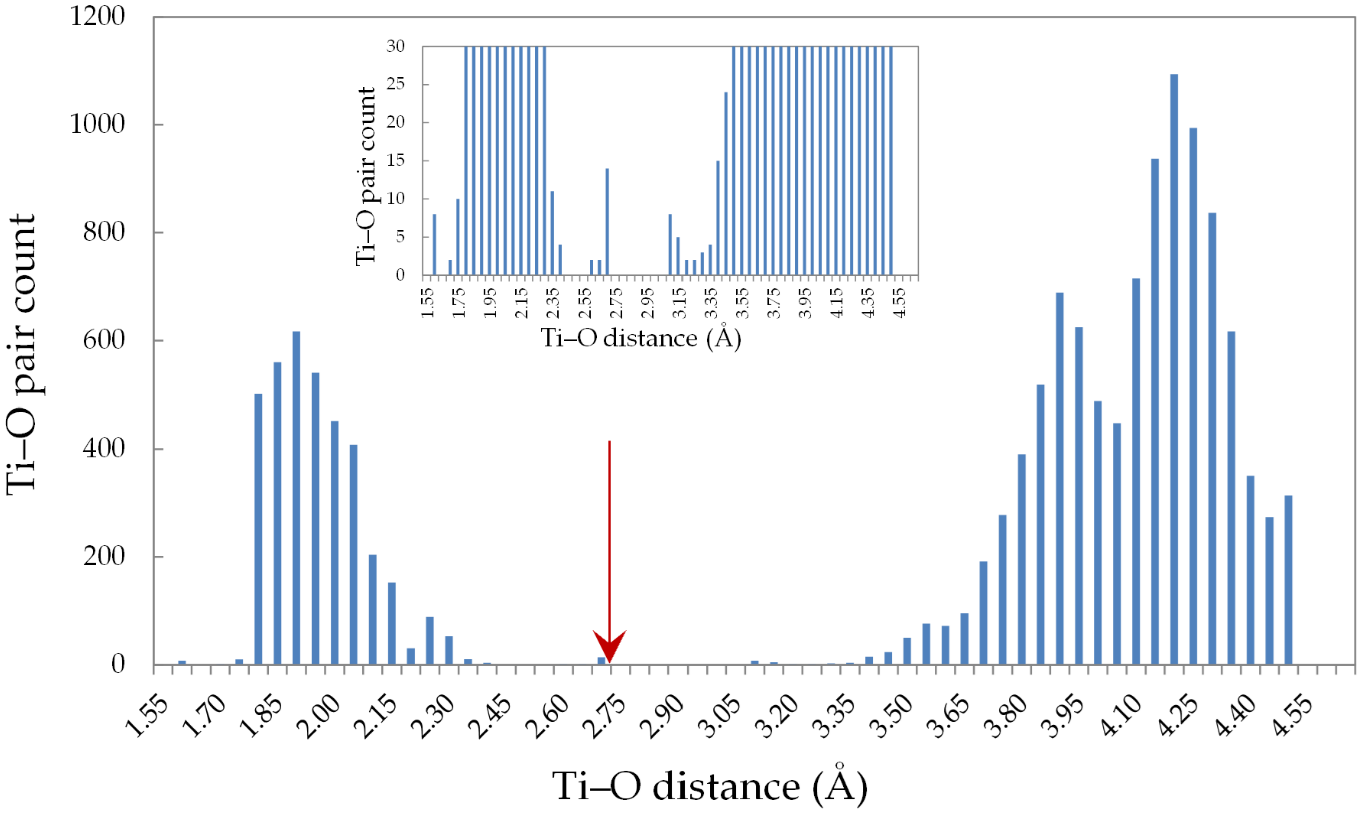

Figure S1. Distribution of Ti–O distances across the nine optimized anatase-derived TiO_2 nanoclusters. The histogram includes all 13,780 Ti–O pairs with $d_{Ti\text{-}O} \leq 4.50$ Å, including pairs involving hydroxyl O atoms, using a bin width of 0.05 Å. The inset shows details of the low count region surrounding the coordination cutoff. The red arrow marks the adopted cutoff of 2.70 Å. The longest included Ti–O contact is 2.674 Å and the shortest excluded contact is 3.063 Å; consequently, the Ti coordination counts remain unchanged when the cutoff is increased from 2.70 to 3.00 Å.

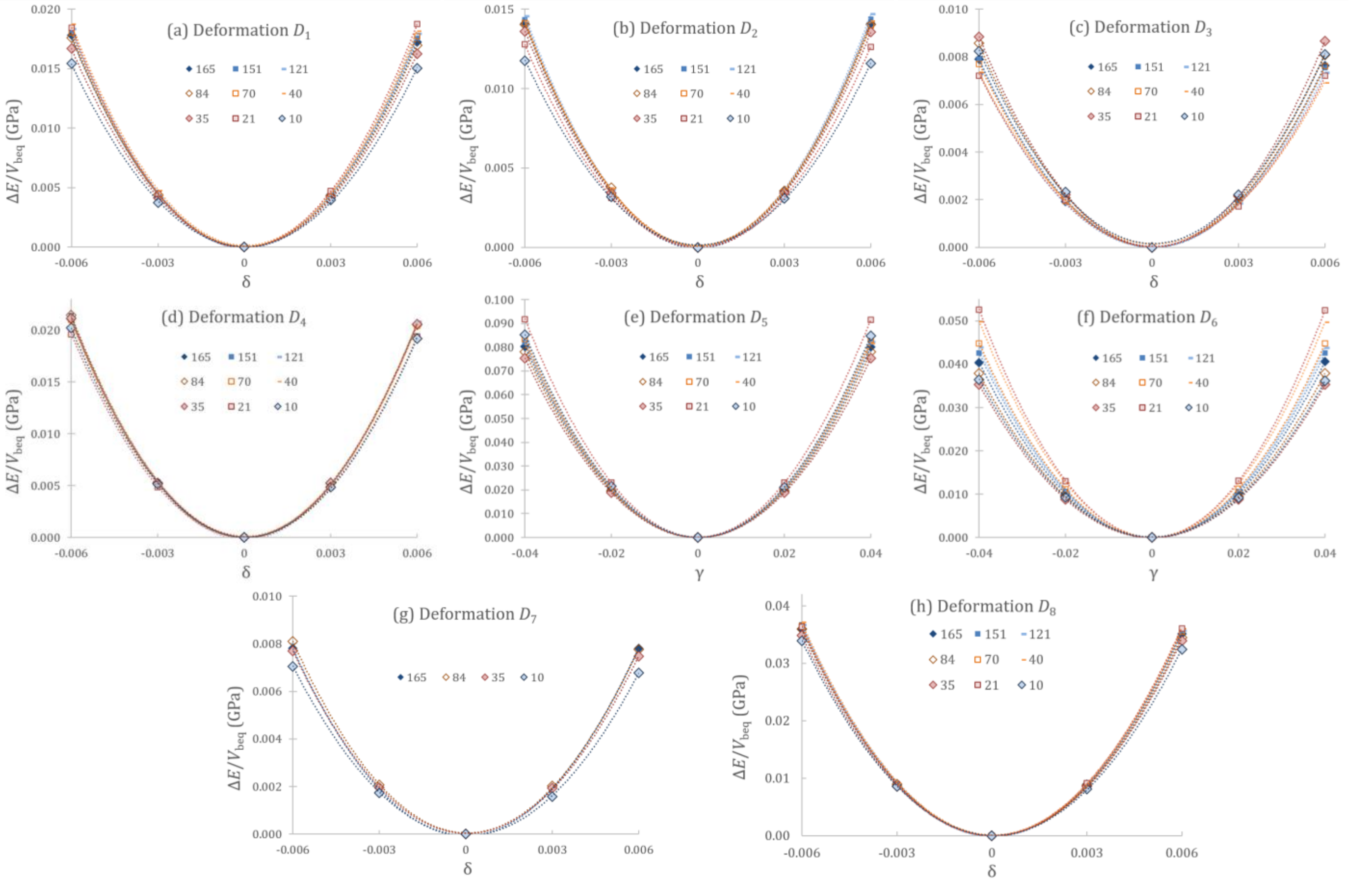

Figure S2. Strain-energy density, the total-energy change divided by the bulk-equivalent volume V_{beq}, as a function of the applied strain for the eight homogeneous deformation modes D_1–D_8 and the nine anatase-derived TiO_2 nanoclusters. The normal-strain modes were sampled at δ = 0, ±0.003 and ±0.006, the shear modes D_5 and D_6 at γ = 0, ±0.02 and ±0.04; every geometry was strained affinely and its energy evaluated without further relaxation of the internal coordinates (clamped-ion). Symbols are the calculated points relative to the unstrained cluster and dotted lines are the least-squares quadratic fits of Eq. (15), whose curvatures $2q_2$ are listed in Table S1. D_7 probes C_{11} directly and was applied to the bipyramidal clusters only, which is why panel (g) shows four curves rather than nine. Because all curves are drawn per bulk-equivalent volume, the vertical spread within a panel reflects differences in curvature per TiO_2 unit rather than differences in cluster size.

Table S1. Energy curvatures obtained from the quadratic fits of Eq. (15) for every cluster and deformation mode: $2q_2$ in eV, its standard error from the least-squares covariance matrix, the intensive curvature per TiO_2 unit $2q_2/n$, the coefficient of determination R^2, and the relative difference between the curvature obtained from the inner strain pair and that from the outer pair. The last column reports the signed symmetric difference between the quadratic coefficients obtained from the inner and outer strain pairs, $200\times(q_{2,\mathrm{inner}} - q_{2,\mathrm{outer}})/(q_{2,\mathrm{inner}} + q_{2,\mathrm{outer}})$. The inner and outer pairs are $\delta = \pm0.003$ and ±0.006 for normal deformations, and $\gamma = \pm0.02$ and ±0.04 for shear deformations. Its largest absolute values are 11.0% for D_3 at $n = 40$ and 10.2% for D_3 at $n = 10$, while the median absolute difference is 0.8%.

n	Morphology	Mode	$2q_2$ (eV)	SE (eV)	$2q_2/n$ (eV)	R^2	inner vs outer (%)
165	bipyramidal	D1	34193.8	511.4	207.24	0.99955	-1.8
165	bipyramidal	D2	27606.6	210.2	167.31	0.99988	-3
165	bipyramidal	D3	15118	172.9	91.62	0.99974	0.2
165	bipyramidal	D4	40453.5	436	245.17	0.99977	0.6
165	bipyramidal	D5	3514.2	2.3	21.3	1	-0.3
165	bipyramidal	D6	1777.6	2.2	10.77	1	-0.7
165	bipyramidal	D7	15349.8	220.4	93.03	0.99959	-5.7
165	bipyramidal	D8	69134.2	533.1	419	0.99988	0.3
151	2-layer cut	D1	31930.3	253.1	211.46	0.99987	0.1
151	2-layer cut	D2	25638.8	24.1	169.79	1	-0.5
151	2-layer cut	D3	13783.2	144.4	91.28	0.99978	-0.1
151	2-layer cut	D4	37429.8	230.4	247.88	0.99992	1
151	2-layer cut	D5	3295.4	1.2	21.82	1	-0.1
151	2-layer cut	D6	1709	0.4	11.32	1	-0.1
151	2-layer cut	D8	64392.1	422.9	426.44	0.99991	0
121	4-layer cut	D1	26057.5	385.8	215.35	0.99956	-3.1
121	4-layer cut	D2	20940.8	15.8	173.06	1	0
121	4-layer cut	D3	10445.8	5.4	86.33	1	0.2
121	4-layer cut	D4	30296	304.3	250.38	0.9998	-5.6
121	4-layer cut	D5	2646.2	3.4	21.87	1	0.2
121	4-layer cut	D6	1413.6	5.9	11.68	0.99996	-1.6
121	4-layer cut	D8	51872.5	270.1	428.7	0.99995	-2.4

Table S1. (contd.).

n	Morphology	Mode	$2q_2$ (eV)	SE (eV)	$2q_2/n$ (eV)	R^2	inner vs outer (%)
84	bipyramidal	D1	17093.1	123.1	203.49	0.9999	1.4
84	bipyramidal	D2	13836.2	145.4	164.72	0.99978	4.3
84	bipyramidal	D3	8229.4	152.1	97.97	0.99932	1.9
84	bipyramidal	D4	20842.9	200.4	248.13	0.99982	-0.2
84	bipyramidal	D5	1741	0.7	20.73	1	0.2
84	bipyramidal	D6	844.6	0.8	10.05	1	0.4
84	bipyramidal	D7	7831.2	69.3	93.23	0.99984	3.3
84	bipyramidal	D8	34848.8	246.4	414.87	0.9999	1
70	2-layer cut	D1	14777.8	124.5	211.11	0.99986	0.7
70	2-layer cut	D2	11605	17.8	165.79	1	0.8
70	2-layer cut	D3	6318.8	11.5	90.27	0.99999	0.8
70	2-layer cut	D4	17119.4	180.7	244.56	0.99978	-0.2
70	2-layer cut	D5	1550.1	0.8	22.14	1	0.2
70	2-layer cut	D6	832.6	1	11.89	1	0.3
70	2-layer cut	D8	29774.5	263.2	425.35	0.99984	-0.5
40	4-layer cut	D1	8668.1	123.6	216.7	0.99959	4.4
40	4-layer cut	D2	6700.5	52.7	167.51	0.99988	4.2
40	4-layer cut	D3	3312.7	97.9	82.82	0.99826	11
40	4-layer cut	D4	9744.5	134	243.61	0.99962	4.1
40	4-layer cut	D5	864.3	2.1	21.61	0.99999	0.9
40	4-layer cut	D6	528.5	1.1	13.21	0.99999	1.2
40	4-layer cut	D8	17225.2	183	430.63	0.99977	2.4

Table S1. (contd.).

n	Morphology	Mode	$2q_2$ (eV)	SE (eV)	$2q_2/n$ (eV)	R^2	inner vs outer (%)
35	bipyramidal	D1	6807.2	54.6	194.49	0.99987	0.6
35	bipyramidal	D2	5608.9	4.5	160.26	1	0.4
35	bipyramidal	D3	3617.4	12.5	103.35	0.99998	0.1
35	bipyramidal	D4	8614.6	60.4	246.13	0.9999	1.4
35	bipyramidal	D5	700	0.2	20	1	-0.2
35	bipyramidal	D6	328.1	0.1	9.37	1	-0.1
35	bipyramidal	D7	3122.9	21.2	89.23	0.99991	2.5
35	bipyramidal	D8	14214.5	78.6	406.13	0.99994	0.1
21	2-layer cut	D1	4617.6	16.2	219.89	0.99998	-1.1
21	2-layer cut	D2	3156.4	23.6	150.31	0.99989	-0.7
21	2-layer cut	D3	1778.9	32.4	84.71	0.99934	2.8
21	2-layer cut	D4	4841.4	15.2	230.54	0.99998	-0.4
21	2-layer cut	D5	510.8	0.8	24.32	0.99999	0.7
21	2-layer cut	D6	293.4	0.6	13.97	0.99999	-0.8
21	2-layer cut	D8	8978.9	42.5	427.57	0.99996	0
10	bipyramidal	D1	1795.9	23.8	179.59	0.99965	1.3
10	bipyramidal	D2	1364.5	18.5	136.45	0.99963	7.1
10	bipyramidal	D3	949.9	19	94.99	0.9992	10.2
10	bipyramidal	D4	2322.5	12.9	232.25	0.99994	1.7
10	bipyramidal	D5	225.7	0.4	22.57	0.99999	0.1
10	bipyramidal	D6	96.1	0.4	9.61	0.99997	2.1
10	bipyramidal	D7	821.9	7	82.19	0.99986	-4.5
10	bipyramidal	D8	3905.6	18.6	390.56	0.99995	1.2

D_7 was applied to the bipyramidal clusters only. Strains: $\delta = \pm 0.003$ and ± 0.006 for D_1–D_4, D_7, D_8; $\gamma = \pm 0.02$ and ± 0.04 for D_5 and D_6.

Table S2. Linear dimensions and reference volumes of the optimized clusters. L_x, L_y and L_z are the extents along the cluster-fixed axes. The three conventions are the bulk-equivalent volume $V_{beq} = n \cdot v_{anat}$ with $v_{anat} = 34.06$ Å^3 per TiO_2 formula unit, the core volume V_{core} given by the convex hull of the TiO_2-framework atoms, and the total volume V_{tot} given by the convex hull of all atoms including the hydroxyl groups; for the stoichiometric bipyramids, which carry no passivation, $V_{core} = V_{tot}$. The two ratios in the last columns are the factors that rescale every dimensional coefficient between conventions through Eqs. (17)–(19): V_{core}/V_{beq} varies from 0.36 to 0.77 across the series, which is why the choice of convention changes the apparent size trend.

n	Morphology	L_x (Å)	L_y (Å)	L_z (Å)	V_{beq} (Å^3)	V_{core} (Å^3)	V_{tot} (Å^3)	V_{core}/V_{beq}	V_{core}/V_{tot}
165	bipyramidal	15.937	16.043	39.29	5619.9	4342	4342	0.7726	1
151	2-layer cut	15.927	16.045	25.283	5143.1	3798.2	4209.9	0.7385	0.9022
121	4-layer cut	15.839	16.048	15.837	4121.3	2896.6	3398.7	0.7029	0.8523
84	bipyramidal	11.998	11.998	32.322	2861	1941.8	1941.8	0.6787	1
70	2-layer cut	12.351	12.039	15.696	2384.2	1532.2	1819.8	0.6427	0.842
40	4-layer cut	12.51	11.479	6.574	1362.4	719.8	1193.5	0.5284	0.6031
35	bipyramidal	8.212	8.231	22.665	1192.1	667.8	667.8	0.5602	1
21	2-layer cut	9.059	8.226	6.707	715.3	331.4	576.4	0.4634	0.575
10	bipyramidal	4.825	5.156	10.278	340.6	123.2	123.2	0.3617	1

Table S3. Effective elastic coefficients and Hill moduli of the nine clusters under the two convex-hull conventions, in GPa, with Poisson's ratio and the universal anisotropy index. The bulk-equivalent values are given in Tables 3 and 4 of the main text. All three sets come from the same energy curvatures and differ only by the factor listed in Table S2, so ν, A^{U} and every ratio between coefficients of the same cluster are identical in all three; the dimensional quantities are not. Comparing these values with the bulk-equivalent ones shows the full size of the normalization effect: B_H spans 200.6–227.1 GPa under V_{beq} against 281.9–554.7 GPa under V_{core}.

n	Morphology	Convention	C_{11} (GPa)	C_{12} (GPa)	C_{13} (GPa)	C_{33} (GPa)	C_{44} (GPa)	C_{66} (GPa)	B_H (GPa)	G_H (GPa)	E (GPa)	ν	A^U
165	bip		570.1	60.77	182.38	557.84	129.67	65.59	281.93	136.56	352.73	0.2915	1.0847
151	2-lc		607.1	66.35	195.18	581.4	139.01	72.09	299.88	145.98	376.8	0.2906	1.0099
121	4-lc		649.89	70.75	224.02	577.78	146.37	78.19	323.16	151.72	393.57	0.297	0.9457
84	bip		637.98	67.18	201.36	679	143.64	69.68	318.93	153.45	396.71	0.2927	1.259
70	2-lc	V_{core}	689.68	82.94	219.84	660.72	162.09	87.06	341.75	169.61	436.6	0.2871	0.8923
40	4-lc		855.15	109.48	288.17	737.32	192.37	117.63	424.04	204.2	527.87	0.2925	0.7222
35	bip		744.74	71.88	227.08	867.9	167.94	78.71	373.59	182.28	470.33	0.2902	1.4532
21	2-lc		939.44	176.56	270.44	859.88	246.92	141.84	463.72	248.91	633.4	0.2723	0.5502
10	bip		1027.53	140.27	378.75	1235.36	293.48	125	554.68	275.93	710.06	0.2866	1.0771
165	bip		570.1	60.77	182.38	557.84	129.67	65.59	281.93	136.56	352.73	0.2915	1.0847
151	2-lc		547.73	59.86	176.09	524.54	125.42	65.04	270.55	131.7	339.95	0.2906	1.0099
121	4-lc		553.89	60.3	190.93	492.43	124.75	66.64	275.42	129.31	335.43	0.297	0.9457
84	bip		637.98	67.18	201.36	679	143.64	69.68	318.93	153.45	396.71	0.2927	1.259
70	2-lc	V_{tot}	580.71	69.83	185.1	556.32	136.48	73.3	287.75	142.81	367.61	0.2871	0.8923
40	4-lc		515.76	66.03	173.8	444.7	116.02	70.95	255.75	123.16	318.37	0.2925	0.7222
35	bip		744.74	71.88	227.08	867.9	167.94	78.71	373.59	182.28	470.33	0.2902	1.4532
21	2-lc		540.22	101.53	155.52	494.47	141.99	81.56	266.66	143.13	364.23	0.2723	0.5502
10	bip		1027.53	140.27	378.75	1235.36	293.48	125	554.68	275.93	710.06	0.2866	1.0771

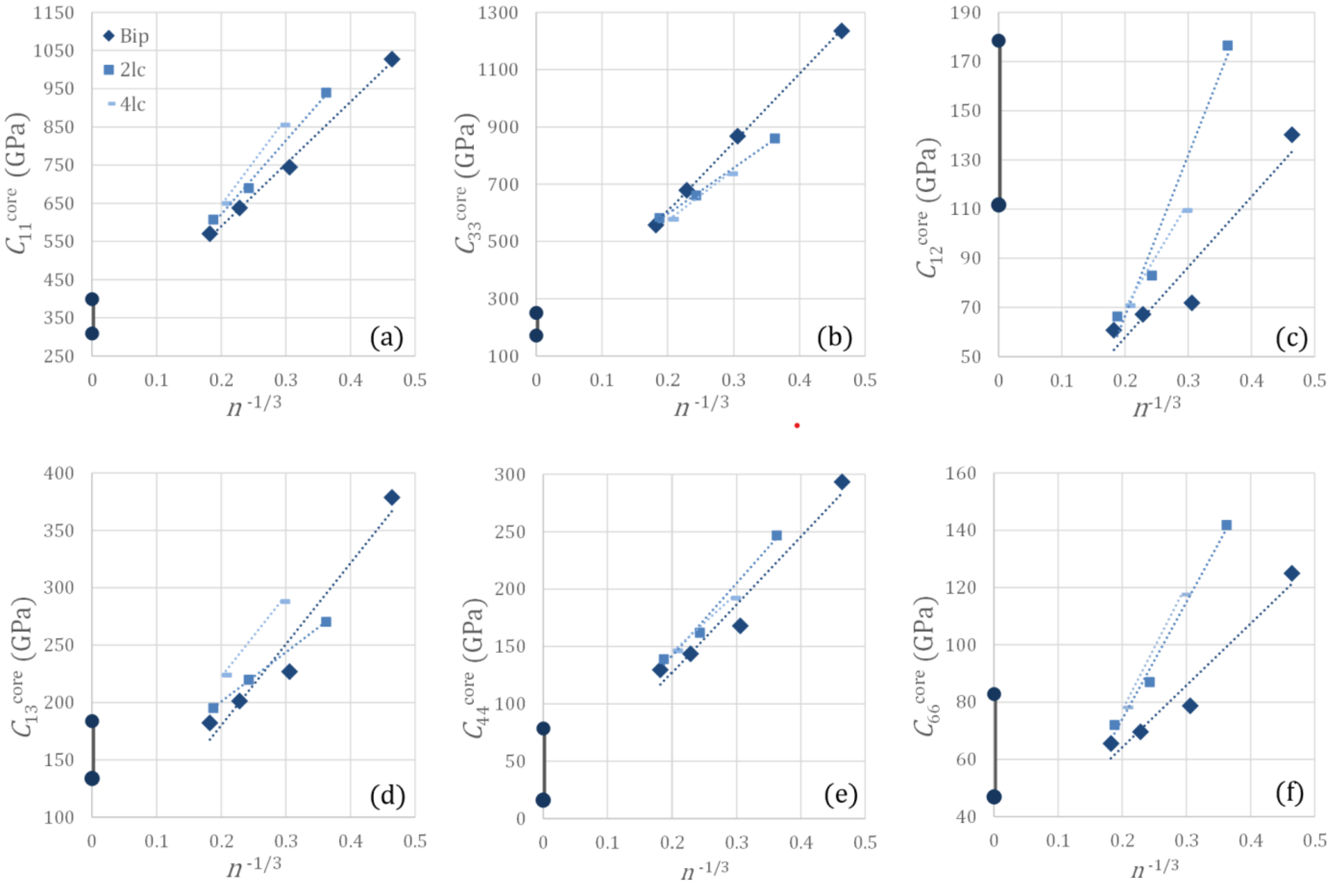

Figure S3. Effective elastic coefficients of the anatase-derived TiO_2 nanoclusters under the core convex-hull convention as a function of inverse cluster size $n^{-1/3}$. Every coefficient now rises steeply toward small clusters. Diamonds, squares, and rectangles denote bipyramidal, 2-layer-cut, and 4-layer-cut clusters, respectively. Vertical bars at $n^{-1/3}\rightarrow 0$ span the range of published DFT values for crystalline anatase (Refs. [23,26-35]). Dotted lines show linear least-squares fits across the nine clusters. The contrast with Figure 3 illustrates the importance of the volume normalization in the calculation of effective elastic coefficients.

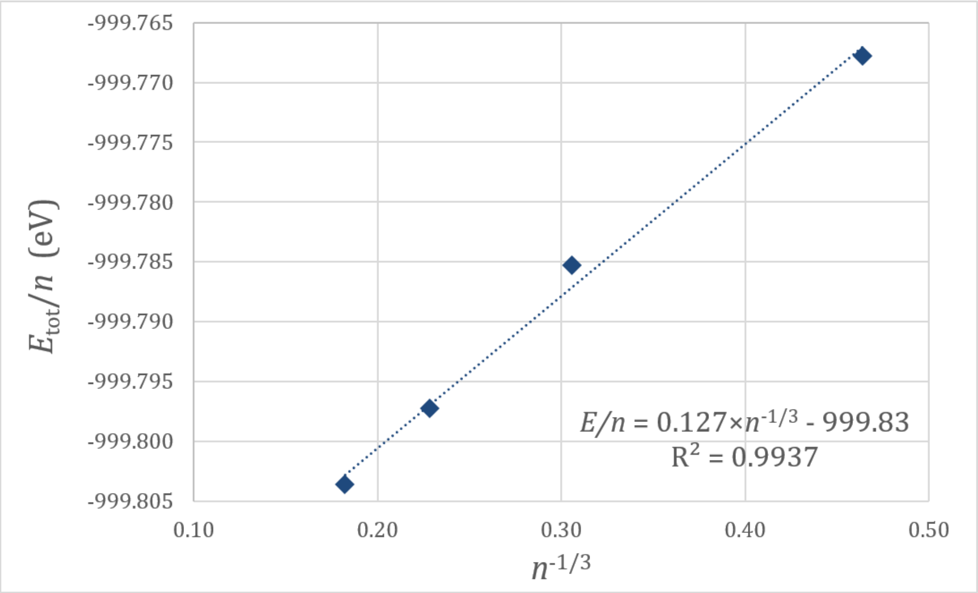

Figure S4. Total energy per TiO_2 unit of the four stoichiometric bipyramidal clusters as a function of inverse cluster size, $n^{-1/3}$. The five cut clusters are hydroxylated, $(TiO_2)_n(H_2O)_m$ with $m = n_{OH}/2$, and are omitted because their energies are not comparable per Ti atom with the stoichiometric set. The dotted line is a linear least-squares fit, $E/n = E_\infty + a \cdot n^{-1/3}$, with $a = 0.127 \pm 0.007$ eV and $E_\infty = -999.826$ eV ($R^2 = 0.9937$, $p = 0.0031$); the residuals are below 2 meV per TiO_2 unit. Leaving out any single cluster shifts the intercept by at most 0.005 eV but the slope by up to 0.022 eV, the largest change coming from $n = 10$, in which 80% of the Ti atoms are four-coordinate.

Table S4. Structural and electronic descriptors of the optimized clusters: the mean Ti–O distance and its standard deviation, the mean O–Ti–O angle and its standard deviation, the fractions f_4–f_6 of Ti atoms with coordination numbers four to six, the aspect ratio *AR*, and the HOMO–LUMO gap from the Kohn–Sham orbital energies. Coordination numbers were assigned with the Ti–O distance cutoff defined in Section 2.5 and examined in Section 3.1. These are the descriptors correlated with the elastic and electronic properties in Sections 3.6 and 3.7. No Ti atom has a coordination number below four under the adopted definition.

n	Morphology	$\langle d_{Ti-O} \rangle$ (Å)	σ_{Ti-O} (Å)	$\langle \theta_{O-Ti-O} \rangle$ (deg)	$\sigma \langle \theta_{O-Ti-O} \rangle$ (deg)	f_4	f_5	f_6	*AR*	Gap (eV)
165	bipyramidal	1.939	0.132	105.64	29.43	0.055	0.515	0.430	2.457	4.06
151	2-layer cut	1.938	0.117	105.52	29.14	0.046	0.510	0.444	1.582	4.052
121	4-layer cut	1.934	0.111	105.31	28.38	0.058	0.537	0.405	0.993	4.088
84	bipyramidal	1.94	0.162	105.93	29.78	0.071	0.619	0.310	2.694	4.078
70	2-layer cut	1.928	0.12	105.42	27.94	0.114	0.571	0.314	1.287	4.206
40	4-layer cut	1.91	0.107	104.76	25.01	0.175	0.725	0.100	0.548	4.271
35	bipyramidal	1.936	0.198	106.34	30.03	0.171	0.686	0.143	2.757	4.095
21	2-layer cut	1.884	0.115	105.15	21.11	0.476	0.524	0.000	0.776	4.411
10	bipyramidal	1.875	0.182	106.23	21.46	0.800	0.200	0.000	2.06	4.529

Table S5. Statistics for every correlation quoted in the main text, calculated using all nine clusters where the descriptors are defined, or seven clusters for descriptors involving 6-coordinate Ti. Pearson coefficients *r* are accompanied by two-sided unadjusted *p* values. Spearman coefficients *ρ* and their two-sided *p* values are reported as rank-based robustness measures and were not subjected to multiple-comparison adjustment. The leave-one-out columns give the minimum and maximum Pearson *r* obtained after omitting one cluster. The *q* values were obtained by applying the Benjamini–Hochberg procedure to the Pearson *p* values within each predefined descriptor family for the same target. The last two columns give Pearson coefficients within the bipyramidal and cut-cluster subsets. With nine clusters drawn from three parent families, and each cut cluster paired to its parent, the analysis is exploratory.

Reported in	Descriptor	Target	*r*	*p*	*ρ*	*p* Spearman	LOO min *r*	LOO max *r*	*q*	*r* bip (4 clusters)	*r* cut (5 clusters)
**1. Structure → elastic**											
§3.4, Table 5, Fig. 4a	$n^{-1/3}$	B_H^{beq}	-0.781966	0.012780	-0.633333	0.067086	-0.859845	-0.480563	0.037717	-0.992377	-0.672050
§3.4, Table 5, Fig. 4b	$n^{-1/3}$	B_H^{core}	0.979999	0.000004	0.966667	0.000022	0.953450	0.989167	0.000033	0.994118	0.981605
§3.4, Table 5, Fig. 4c	$n^{-1/3}$	B_H^{tot}	0.742695	0.021886	0.250000	0.516490	0.141101	0.896283	0.050986	0.994118	-0.432622
§3.4, Table 5,	f_4	B_H^{beq}	-0.777805	0.013595	-0.566667	0.111633	-0.859669	-0.384714	0.037717	-0.939722	-0.776509
§3.4, Table 5,	f_4	B_H^{core}	0.949439	0.000091	0.966667	0.000022	0.895414	0.984827	0.000273	0.983762	0.899020
§3.4, Table 5,	f_4	B_H^{tot}	0.765393	0.016229	0.166667	0.668231	-0.123030	0.935196	0.050986	0.983762	-0.335705
§3.4, Fig. 4d	$\sigma_{Ti–O}$	B_H^{beq}	-0.825896	0.006088	-0.800000	0.009628	-0.882547	-0.802753	0.037717	-0.705813	-0.558964
§3.6, Fig. 6a	$\langle\theta_{O–Ti–O}\rangle$	C_{33}^{beq}	0.940366	0.000161	0.983333	0.000002	0.923849	0.978803	0.001447	0.758378	0.920958
§3.6, Fig. 6b	$\langle\theta_{O–Ti–O}\rangle$	C_{66}^{beq}	-0.928363	0.000302	-0.966667	0.000022	-0.988829	-0.909722	0.002717	-0.993386	-0.727737
§3.6, Fig. 6c	*AR*	A^U	0.907596	0.000721	0.966667	0.000022	0.889566	0.932301	0.006487	0.809579	0.767430
§3.6, Fig. 6d	$\langle\theta_{O–Ti–O}\rangle$	C_{33}/C_{11}	0.954365	0.000064	0.950000	0.000088	0.936925	0.967459	0.000288	0.957100	0.861406
§3.6, Fig. 6e	$\sigma_{Ti–O}$	C_{33}/C_{11}	0.968130	0.000018	0.983333	0.000002	0.965514	0.977052	0.000166	0.923391	0.968891
§3.6, Fig. 6f	*AR*	C_{33}/C_{11}	0.784153	0.012366	0.866667	0.002495	0.713857	0.890285	0.037097	-0.311018	0.882271
§3.6 text, Table S5	$\langle\theta_{O–Ti–O}\rangle$	A^U	0.857600	0.003112	0.916667	0.000507	0.787355	0.919755	0.014006	0.551416	0.659111
§3.6 text, Table S5	σ(Ti–O)	A^U	0.803309	0.009113	0.833333	0.005266	0.662147	0.863987	0.027340	0.635818	0.273490
**2. Structure → electronic**											
§3.7, Fig. 7a	$n^{-1/3}$	ΔE_HL	0.920381	0.000434	0.916667	0.000507	0.827871	0.982156	0.000975	0.935160	0.992871
§3.7, Fig. 7b	f_4	ΔE_HL	0.938989	0.000174	0.966667	0.000022	0.898320	0.962866	0.000522	0.995817	0.932629
§3.7, Fig. 7c	$\langle d_{Ti–O}\rangle$	ΔE_HL	-0.986111	0.000001	-0.916667	0.000507	-0.994359	-0.975506	0.000009	-0.998968	-0.968273
**3. Electronic → elastic**											
§3.7, Fig. 8a	$\langle Q_{Ti}\rangle_6$	C_{11}^{beq}	0.959326	0.000627	0.928571	0.002519	0.924032	0.982299	0.002299	0.999930	0.832600
§3.7, Fig. 8b	$\langle Q_{Ti}\rangle_6$	B_H^{beq}	0.921410	0.003187	0.928571	0.002519	0.835188	0.968201	0.011684	0.987431	0.804883
§3.7, Fig. 8c	$\langle MBO\rangle_5$	C_{66}^{beq}	0.902255	0.000873	0.966667	0.000022	0.869544	0.955939	0.009599	0.451262	0.934924
§3.7, Fig. 8d	$\langle Q_{Ti}\rangle_6$	C_{33}/C_{11}	-0.956385	0.000745	-0.964286	0.000454	-0.966882	-0.955968	0.008198	-0.985713	-0.931336
§3.7, Fig. 8e	$\langle MBO\rangle_5$	C_{33}/C_{11}	-0.885354	0.001499	-0.933333	0.000236	-0.933705	-0.798393	0.008246	-0.720635	-0.749850
§3.7, Fig. 8f	$\langle Q_{Ti}\rangle_4$	A^U	0.877614	0.001870	0.966667	0.000022	0.788189	0.909984	0.018944	0.925572	0.910775
§3.7 text, Table S5	$\langle MBO\rangle_4$	A^U	-0.818236	0.007022	-0.916667	0.000507	-0.902801	-0.682273	0.019311	0.463081	-0.963436

Table S6. Coordination-resolved mean Mayer Ti–O bond orders $\langle \mathrm{MBO} \rangle_k$ and mean Hirshfeld charges $\langle Q \rangle_k$ of the Ti atoms in coordination class k = 4, 5, and 6, with the standard deviation within each class, at the same level of theory as the structural calculations. Bond orders are grouped by the coordination number of the Ti atom of the bond. A dash marks a coordination class that is absent from the cluster.

n	Morphology	$\langle \mathrm{MBO} \rangle_6$	σ	$\langle \mathrm{MBO} \rangle_5$	σ	$\langle \mathrm{MBO} \rangle_4$	σ	$\langle Q \rangle_6$ (e)	σ	$\langle Q \rangle_5$ (e)	σ	$\langle Q \rangle_4$ (e)	σ
165	bipyramidal	0.5937	0.0444	0.6594	0.0452	0.8046	0.0526	0.6741	0.0052	0.7368	0.0121	0.7772	0.026
151	2-layer cut	0.589	0.0467	0.6808	0.0453	0.8237	0.0441	0.6756	0.0044	0.7321	0.0081	0.768	0.0216
121	4-layer cut	0.5982	0.0392	0.7177	0.063	0.8246	0.0446	0.6772	0.0056	0.7288	0.0065	0.7708	0.022
84	bipyramidal	0.617	0.02	0.6539	0.0426	0.8203	0.1494	0.6679	0.004	0.7373	0.0124	0.8279	0.0409
70	2-layer cut	0.6125	0.0206	0.6908	0.0435	0.8418	0.0001	0.6738	0.0082	0.7276	0.0078	0.7647	0.0205
40	4-layer cut	0.5815	0.0018	0.7585	0.0576	0.9154	0.1226	0.6783	0.0026	0.721	0.0099	0.764	0.0219
35	bipyramidal	0.6201	0.012	0.6496	0.0377	0.8182	0.1522	0.6543	0.0077	0.7362	0.015	0.8341	0.0363
21	2-layer cut	—	—	0.7667	0.0559	0.9292	0.1038	—	—	0.7137	0.0244	0.758	0.0169
10	bipyramidal	—	—	0.5649	0	0.8189	0.0413	—	—	0.7177	0.0003	0.7825	0.0182

Table S7. Targeted tests of the tetragonal transverse equalities for the three 2-layer-cut clusters. Coefficients are in GPa under core-hull normalization. $A = (C_{11}+C_{22})/2$ is obtained from D_1 and D_2. C_{22} and C_{55} are obtained from the added y-uniaxial and *xz*-shear curves. $C_{11} = 2A-C_{22}$. The first percentage uses the transverse mean as denominator; the shear percentage uses C_{44}. Relative quantities are independent of the volume convention for a fixed cluster. Original and added tests used the same optimized reference geometry, functional, numerical settings and strain amplitudes.

n	A	C_{11}	C_{22}	$100(C_{22}-C_{11})/A$ (%)	C_{44}	C_{55}	$100(C_{55}/C_{44}-1)$ (%)
151	607.10	604.34	609.85	+0.91	139.01	139.12	+0.08
70	689.68	684.56	694.80	+1.48	162.09	159.33	-1.70
21	939.46	849.68	1029.25	+19.11	246.91	217.92	-11.74

Table S8. Reconstructed normal-strain block and measured shear diagonal entries, in GPa under core-hull normalization. The normal-strain block was reconstructed using Eq. (S1). The remaining normal–shear and shear–shear coupling terms were not measured and are retained as zero in the expanded sensitivity matrix. This is not a complete elastic-tensor determination or an assertion of exact orthorhombic symmetry.

n	C_{11}	C_{22}	C_{33}	C_{12}	C_{13}	C_{23}	C_{44}	C_{55}	C_{66}
151	604.34	609.85	581.40	66.35	196.56	197.39	139.01	139.12	72.09
70	684.56	694.80	660.72	82.94	222.40	231.30	162.09	159.33	87.06
21	849.68	1029.25	859.89	176.58	315.34	308.79	246.91	217.92	141.84

Table S9. Aggregate quantities from the original six-coefficient form and expanded nine-coefficient sensitivity matrix. B_H and G_H are in GPa under core-hull normalization; A^U is dimensionless. Percentage changes are 100(expanded/original−1). ΔA^U is expanded minus original in absolute units. Both matrices were reconstructed from the same freely fitted curves; minor differences from the original rounded tables follow from refitting. The aggregate quantities were calculated using Eqs. (S2)–(S7). These point-estimate differences are not bounds on unmeasured couplings or on errors outside the reconstruction.

n	B_H original	B_H expanded	ΔB_H (%)	G_H original	G_H expanded	ΔG_H (%)	A^U original	A^U expanded	ΔA^U
151	299.882	300.629	0.249	145.980	145.809	-0.117	1.009911	1.007665	-0.002246
70	341.748	344.676	0.857	169.605	168.310	-0.764	0.892283	0.887369	-0.004914
21	463.740	480.845	3.688	248.908	238.098	-4.343	0.550217	0.557265	+0.007047

Table S10. Fit diagnostics for the added y-uniaxial and xz-shear modes. Curvatures $2q^2$ and their residual-based standard errors are in eV, with dimensionless strain or engineering shear as the independent variable. The signed inner/outer measure is $200(q^2,\text{inner}-q^2,\text{outer})/(q^2,\text{inner}+q^2,\text{outer})$. The curves were fitted as $\Delta E = q_0 + q_1\ \varepsilon + q_2\ \varepsilon^2$, with intercept and slope retained. Each fit has five observations and two residual degrees of freedom. Standard errors characterize the fitted residuals and are not complete electronic-structure uncertainties.

n	Mode	$2q_2$ (eV)	SE (eV)	R^2	Inner/outer (%)
151	Y	14457.5575	79.8143	0.9999391	-2.42
151	XZ shear	3297.9762	1.2758	0.9999997	-0.13
70	Y	6644.6392	48.2753	0.9998944	-3.61
70	XZ shear	1523.7482	0.8523	0.9999994	+0.30
21	Y	2129.2574	13.4961	0.9999197	-3.35
21	XZ shear	450.8229	0.8575	0.9999928	-0.16

Table S11. Basis-and-geometry sensitivity for the n = 10 cluster. SVP and TZVP reference structures were optimized separately with B3LYP; the TZVP calculation used TightSCF. The test therefore includes geometry reoptimization and does not isolate a basis effect at fixed coordinates. The fitted mode curvatures and standard errors are in eV. Their percentage changes equal those of the corresponding normalized quadratic coefficients when the same reference-volume factor is used. D_3 and D_6 changes also equal those of C_{33} and C_{66} under that convention. The D_8 curvature is a deformation-path result and is not the Hill bulk modulus. Fit uncertainty measures have the same definitions and limits as Table S10.

Mode	Basis	$2q_2$ (eV)	SE (eV)	SE (%)	R^2	Inner/outer (%)	TZVP/SVP – 1 (%)
D8 isotropic	SVP	3905.5911	18.6495	0.48	0.9999545	+1.15	reference
D8 isotropic	TZVP	3755.7953	79.6446	2.12	0.9991025	-2.41	-3.84
D3 axial	SVP	949.9026	19.0416	2.00	0.9991971	+10.24	reference
D3 axial	TZVP	916.1211	18.2617	1.99	0.9992090	-7.40	-3.56
D6 basal shear	SVP	96.1167	0.3745	0.39	0.9999696	+2.13	reference
D6 basal shear	TZVP	97.2816	1.2631	1.30	0.9996630	-3.07	+1.21

Table S12. Raw total energies and differences for the basis-and-geometry comparison. Each basis uses its own optimized reference and undeformed energy. Normal strains are 0, ±0.003 and ±0.006; engineering shear is 0, ±0.02 and ±0.04. ΔE values are in eV. Absolute energies across the two bases were not subtracted to infer stiffness.

Basis	Mode	Strain	E (Hartree)	ΔE (eV)
SVP	D8 isotropic	+0.006	-9997.6753433258	0.06881614
SVP	D8 isotropic	+0.003	-9997.6772356562	0.01732321
SVP	D8 isotropic	+0.000	-9997.6778722724	0.00000000
SVP	D8 isotropic	-0.003	-9997.6771999940	0.01829363
SVP	D8 isotropic	-0.006	-9997.6752256451	0.07201840
TZVP	D8 isotropic	+0.006	-10000.5721418520	0.06538258
TZVP	D8 isotropic	+0.003	-10000.5739229879	0.01691541
TZVP	D8 isotropic	+0.000	-10000.5745446177	0.00000000
TZVP	D8 isotropic	-0.003	-10000.5739576769	0.01597147
TZVP	D8 isotropic	-0.006	-10000.5719953757	0.06936841
SVP	D3 axial	+0.006	-9997.6772385774	0.01724372
SVP	D3 axial	+0.003	-9997.6776998219	0.00469262
SVP	D3 axial	+0.000	-9997.6778722724	0.00000000
SVP	D3 axial	-0.003	-9997.6776912083	0.00492701
SVP	D3 axial	-0.006	-9997.6772295994	0.01748802
TZVP	D3 axial	+0.006	-10000.5739262934	0.01682546
TZVP	D3 axial	+0.003	-10000.5743864327	0.00430443
TZVP	D3 axial	+0.000	-10000.5745446177	0.00000000
TZVP	D3 axial	-0.003	-10000.5744242732	0.00327474
TZVP	D3 axial	-0.006	-10000.5739631721	0.01582194
SVP	D6 basal shear	+0.040	-9997.6750498494	0.07680204
SVP	D6 basal shear	+0.020	-9997.6771543455	0.01953579
SVP	D6 basal shear	+0.000	-9997.6778722724	0.00000000
SVP	D6 basal shear	-0.020	-9997.6771424935	0.01985830
SVP	D6 basal shear	-0.040	-9997.6750257283	0.07745841
TZVP	D6 basal shear	+0.040	-10000.5716769124	0.07803424
TZVP	D6 basal shear	+0.020	-10000.5738794706	0.01809957
TZVP	D6 basal shear	+0.000	-10000.5745446177	0.00000000
TZVP	D6 basal shear	-0.020	-10000.5738288901	0.01947594
TZVP	D6 basal shear	-0.040	-10000.5717168393	0.07694777

Section S1. *Reconstruction of the expanded stiffness matrix and calculation of aggregate elastic quantities, Eqs. (S1)–(S7)*

Let $k_m = 2q_{2,m}/V_{\text{ref}}$, expressed in stiffness units, and let k_y denote the new y-uniaxial curvature per reference volume. Substitution of the normal deformation modes in Eq. (7) of the main manuscript into the general quadratic energy–strain expression, Eq. (2), without imposing the transverse equalities, gives:

$$\begin{aligned} C_{22} &= k_y, \\ C_{11} &= \frac{k_1 + k_2}{2} - C_{22}, \\ C_{12} &= \frac{k_1 - k_2}{4}, \\ C_{33} &= k_3, \\ C_{13} &= \frac{k_4 - C_{11} - C_{33}}{2}, \\ C_{23} &= \frac{k_8 - k_1 - k_3}{2} - C_{13}. \end{aligned} \tag{S1}$$

The three shear coefficients follow from the respective yz, xz and xy shear curvatures. Engineering shear is $\gamma = 2\,\varepsilon_{ij}$. The original six-coefficient C_{13} differs from the reconstructed entry by $(C_{11}-C_{22})/4$. In particular, the $n = 21$ reconstruction increases C_{13} to approximately 315.3 GPa under core normalization, or 146.1 GPa under bulk-equivalent normalization.

The Voigt and Reuss moduli were calculated from the expanded stiffness matrix $\boldsymbol{C}$ and its inverse $\boldsymbol{S}$ using the general expressions [64]:

$$B_V = \frac{C_{11} + C_{22} + C_{33} + 2(C_{12} + C_{13} + C_{23})}{9}, \tag{S2}$$

$$B_R = \frac{1}{S_{11} + S_{22} + S_{33} + 2(S_{12} + S_{13} + S_{23})}, \tag{S3}$$

$$G_V = \frac{C_{11} + C_{22} + C_{33} - C_{12} - C_{13} - C_{23} + 3(C_{44} + C_{55} + C_{66})}{15}, \tag{S4}$$

$$G_R = \frac{15}{4(S_{11} + S_{22} + S_{33} - S_{12} - S_{13} - S_{23}) + 3(S_{44} + S_{55} + S_{66})}. \tag{S5}$$

The Hill moduli [64] and universal anisotropy index [65] are:

$$B_H = \frac{B_V + B_R}{2}, \qquad G_H = \frac{G_V + G_R}{2}, \tag{S6}$$

$$A^U = 5\frac{G_V}{G_R} + \frac{B_V}{B_R} - 6. \tag{S7}$$
